# Mechanisms of antibiofilm compounds JG-1 and M4 across multiple species: alterations of protein interactions essential to biofilm formation

**DOI:** 10.3389/fcimb.2025.1631575

**Published:** 2025-09-17

**Authors:** Aliyah N. Bennett, Jacob F. Maziarz, Baileigh Laipply, Allysa L. Cole, Katherine J. Woolard, Amy Sorge, Michael J. Zeiler, Roberta J. Melander, Christian Melander, John S. Gunn

**Affiliations:** ^1^ Center for Microbe and Immunity Research, Abigail Wexner Research Institute at Nationwide Children’s Hospital, Columbus, OH, United States; ^2^ Infectious Diseases Institute, The Ohio State University, Columbus, OH, United States; ^3^ Department of Veterinary Biosciences, The Ohio State University, Columbus, OH, United States; ^4^ Department of Chemistry and Biochemistry, University of Notre Dame, Notre Dame, IN, United States; ^5^ Department of Pediatrics, College of Medicine, The Ohio State University, Columbus, OH, United States

**Keywords:** *Salmonella*, antibiofilm, ESKAPE pathogens, thermal proteome profiling, RNAseq

## Abstract

The majority of human bacterial pathogens have the ability to form biofilms *in vivo* on body tissues and implantable medical devices. Biofilm-mediated chronic bacterial infections are difficult to treat due to their recalcitrance to antimicrobials and immune effectors, often requiring invasive surgical intervention to clear the infection. The difficulty in effectively executing these treatment strategies underscores the need for therapeutic agents that specifically target the biofilm state. To this end, we previously identified two small molecules, JG-1 and M4, that *in vitro* effectively inhibit and disperse biofilms of *Salmonella* Typhimurium and members of the ESKAPE pathogen group, including *Enterobacter cloacae*, *Pseudomonas aeruginosa*, and *Acinetobacter baumannii*. In addition to its antibiofilm effects, M4 has a bactericidal effect on *Staphylococcus aureus* and *Enterococcus faecium*. While these compounds have promising utility as antimicrobial agents, their mechanism of action remains unknown. By employing multiple techniques including RNAseq, thermal proteome profiling, and site directed mutagenesis, we identified multiple proteins essential to biofilm formation and evaluated their role in the presence of JG-1 and M4 in mutant and wildtype backgrounds. We report that the JG-1 and M4 actions are influenced by proteins important to biofilm maintenance, including OmpA, OmpC, and TrxA. Compound-bacteria interactions cause transcriptional changes that result in biofilm dispersal, and modulation of other virulence mechanisms, including invasion and motility. Additionally, we report that M4 interacts with *S. aureus* CodY, which promotes cell death, while the specific targets in *S*. Typhimurium and *E. cloacae* remain elusive. Collectively, this study presents an empirical investigation into JG-1 and M4’s mechanism of action in *S*. Typhimurium, *E. cloacae*, and *S. aureus*, and how the antibiofilm compounds disrupt microbial community dynamics, ultimately driving biofilm dispersal or cell death.

## Introduction

Despite the discovery of antibiotics, infections remain a top cause of death with 7.7 million deaths attributed to bacterial infections in 2019 (Ikuta et al., 2022; Murray et al., 2022). Of those, 1.27 million were caused by bacteria resistant to available anitbiotics (Murray et al., 2022; Okeke et al., 2024). Experts agree that new antimicrobial agents are needed to tackle this problem (Laxminarayan et al., 2024). While the majority of efforts have been directed at targeting infections caused by free-swimming (planktonic) bacteria, additional efforts are needed to develop new antimicrobial agents that target chronic or biofilm-mediated infections. Bacterial biofilms present a challenge due to their community-mediated tolerance to many antimicrobials, and resistance to physical removal. It is estimated that up to 65-80% of human infections are biofilm-mediated with 17 million biofilm-associated infections and 550,000 deaths each year in the United States alone (National Institutes of Health, 1997; Hung and Henderson, 2009; Wolcott et al., 2010).

Biofilms are communities of microbes that secrete an extracellular matrix that allows them to adhere to and persist on surfaces. This process begins with planktonic bacteria secreting extra polymeric substances to attach to a surface and each other (Prouty et al., 2002; Gunn et al., 2016). There is great diversity in matrix composition between microbes (Zogaj et al., 2003; Barnhart and Chapman, 2006; Taglialegna et al., 2016; Erskine et al., 2018; Tursi and Tükel, 2018; Serra and Hengge, 2019), but up to 80% of bacteria exist in biofilms (Bacteria and archaea on Earth and their abundance in biofilms), underscoring the ubiquity of this phenotype. Following the early attachment stage, bacteria then replicate or attract other microbes in order to expand this structure into a “mature” biofilm (Bjarnsholt, 2013; Wolcott et al., 2010; Xu et al., 2023). The extracellular matrix and the biofilm lifestyle facilitate bacterial resistance to removal, with the matrix allowing for diffusion of nutrients, signaling, or quorum sensing molecules (Quorum sensing: microbial rules of life; Hammer and Bassler, 2003; Ng and Bassler, 2009; Santajit et al., 2022), and horizontal transfer of genetic elements (Costerton et al., 1999), while limiting the diffusion of offensive substances such as antimicrobial peptides, antibodies, reactive oxygen species, and most antibiotics (Rabin et al., 2015; Ferreira et al., 2024). There is a spectrum of metabolic activity within a biofilm, with the cells at the base of the biofilm generally in a sessile state and bacteria at the periphery highly metabolically active (Crabbé et al., 2019; Malviya et al., 2023; Zhu et al., 2007), and many antibiotics only affect these peripheral, metabolically active cells (Sharma et al., 2019). Bacteria within the biofilm can readily transition between states and release from the biofilm to seek out new sites to colonize (Guilhen et al., 2017).

*Salmonella* form biofilms in diverse environments, from plants, to abiotic surfaces, and in some cases, during human infection. Colonization of plant or food surfaces by non-typhoidal serovars of *Salmonella* (NTS) increases the risk of bacterial contamination during food processing or consumption (Vestby et al., 2009; Yaron and Römling, 2014; Wang et al., 2022; Pérez-Lavalle et al., 2023; Ivers et al., 2024). Following acute human infection, *Salmonella* Typhi, a typhoidal *Salmonella* serovar, can persist in the host by forming biofilms on gallstones. These biofilms are the major facilitator of chronic carriage of the bacteria and intermittently release bacteria into the intestines to be shed into the environment, potentially infecting a new host (Prouty et al., 2002; Crawford et al., 2010a; Gunn et al., 2014; Marshall et al., 2014; Muturi et al., 2024a; Muturi et al., 2024b). Treatment of these chronic carriers is essential to reducing human-to-human spread of *S.* Typhi, however, antibiotics are only moderately successful at clearing carriage (Anderson et al., 1936; Ferreccio et al., 1988; Gotuzzo et al., 1988; Bhandari et al., 2024). The most effective treatment, cholecystectomy, has a cure rate of 85%, but eradication failures are associated with other sites of bacterial colonization, including the liver and biliary tree (Main, 1961; Ristori et al., 1982; Nath et al., 2011; Gunn et al., 2014). Recent global spread of invasive non-typhoidal *Salmonella* serovars (iNTS) (Kim et al., 2024; Raju et al., 2024; Sima et al., 2024; Li et al., 2025; Zheng et al., 2025) and the evidence of human-to-human spread, possibly facilitated by a similar biofilm-mediated chronic carriage (Kering et al; Vasicek and Gunn), underscores the urgency of developing systemic treatment strategies that effectively disrupt *Salmonella* biofilms.

The 2024 World Health Organization report on critical or high priority pathogens for antibacterial drug development includes *S.* Typhi, NTS, and all members of the ESKAPE pathogen group (WHO Bacterial Priority Pathogens List 2024: Bacterial Pathogens of Public Health Importance, to Guide Research, Development, and Strategies to Prevent and Control Antimicrobial Resistance, 2024). The ESKAPE pathogens are a group of human bacteria highlighted for their increasing incidence in health-care associated infections and decline in effective treatments due to spread of novel mechanisms of antimicrobial resistance (Pendleton et al., 2013; Santajit and Indrawattana, 2016; Ma et al., 2019). Importantly, these pathogens, *Enterobacter* spp., *Staphylococcus aureus, Klebsiella pneumoniae, Acinetobacter baumannii, Pseudomonas aeruginosa*, and *Enterococcus faecium*, are responsible for the majority of biofilm infections on indwelling medical devices (Pendleton et al., 2013). The unique challenges presented by biofilms demands strategies designed to target this morphotype in order to more effectively treat chronic infections.

To address this issue we identified antibiofilm agents that target the *Salmonella* biofilm state (Huggins et al., 2018). These small molecules, JG-1 and M4, inhibit and disperse biofilms *in vitro*, and work cooperatively with ciprofloxacin to reduce *Salmonella* biofilm burden in a murine model of chronic typhoidal gallbladder carriage (Sandala et al., 2020; Woolard et al., 2022). We have recently demonstrated that these antibiofilm compounds have broad-spectrum activity in addition to *Salmonella*, including *in vitro* antibiofilm activity against *Enterobacter* spp., *S. aureus, A. baumannii, P. aeruginosa*, and *E. faecium* (Bennett et al., 2023). We also demonstrated that one of these compounds, M4, also has bactericidal activity against *S. aureus* and *E. faecium*. While we have established their efficacy, we seek to understand their mechanism of action in both their antibiofilm and bactericidal activity. Here we present a detailed investigation on the response of affected bacteria to compound treatment, as well as potential protein interactors that facilitate JG-1 and M4’s activity *in vitro*.

## Materials and methods

### Bacterial strains, growth conditions, and compounds

Bacteria used include *Salmonella enterica* subsp. *enterica* serovar Typhimurium 14028, *Staphylococcus aureus* USA300, and *Enterobacter cloacae subsp. cloacae* (Jordan) Hormaeche and Edwards, subsp. nov. ATCC 13047. All single gene deletion mutants of *S. aureus* and where indicated, single and multiple gene deletion mutants of *S.* Typhimurium, were obtained through BEI Resources (NIAID, NIH). See **Supplementary Table 1** for details of individual isolates used in this study (Fitzsimmons et al., 2020; Muller et al., 2009; Porwollik et al., 2014).

Individual bacterial colonies were used to inoculate tryptic soy broth ([TSB]; Fisher Cat No. DF0370-07-5) (*S.* Typhimurium and *S. aureus*) or Luria Bertani broth ([LB]; Fisher Cat No. BP1426-2) (*E. cloacae*) for liquid overnight (O/N) cultures grown at 37°C with aeration in a rolling drum.

*S.* Typhimurium double mutants generated in this study were created using P22HT*int* transduction. In brief, donor mutants (with either a kanamycin [Kan] or chloramphenicol [Cam] insertion in the first gene of interest) were infected with P22HT*int*. The donor P22 lysate was then introduced to a recipient mutant (with a different antibiotic cassette insertion in the second gene of interest) in order to create a double gene-deletion mutant. The double mutant was selected for on LB agar with Kan (45 µg/mL) and Cam (20 µg/mL) and then confirmed phage-free on Evan’s blue uridine (EBU) agar.

The conditions used to grow biofilms for each species were previously described in detail (Sandala and Gunn, 2021; Bennett et al., 2023). In brief, single species biofilms were grown in media alone in 96-well plates (Corning Cat No. 7200656) for 24 hours. After the initial incubation, the spent media was removed and replaced with media containing each respective anti-biofilm compound (diluted from 100 mM stock) or equal volume of vehicle (DMSO). Biofilms were then re-incubated for up to 24 hours prior to analysis.

Identification of compounds M4 and JG-1 were previously described (see **Supplementary Figures 1A, C** for their respective structures) (Sandala et al., 2020; Woolard et al., 2022). HCl-salted samples of M4 and JG-1 were stored as powders, shielded from light, at -20°C. Stock solutions were prepared in DMSO at a concentration of 100 mM and stored at -20°C. All further dilutions were prepared in culture media such that the final DMSO concentration was no greater than 10% (v/v). In experiments where the effects of more than one concentration were tested, the concentration of the vehicle was standardized across all conditions by adding an equal volume of DMSO.

### Biofilm inactivation assay

#### Kinetic assay

Biofilms of a bioluminescent isolate of *S.* Typhimurium (14028lux) (Bennett et al., 2024) were grown for 24 hours in a clear 96-well plate. After incubation, spent media was removed and replaced with an equal volume of 1:20 TSB or 0.5%-10% sodium azide (NaN_3_) (w/v) in 1:20 TSB. The plate was placed in a SpectraMax M3 (VWR) plate reader maintained at 30°C for the duration of the experiment. Relative Luminescence (RLU) of each well as measured every 15 minutes for 16 hours and compared between conditions to determine the minimal duration of treatment and inhibitory concentration (MIC) for NaN_3_ to achieve bacteriostasis of *S.* Typhimurium biofilms.

#### Biofilm inactivation with compound treatment

Biofilms of 14028lux were grown for 24 hours in a clear 96-well plate. After incubation, the supernatant was removed and replaced with an equal volume of 1:20 TSB or 6% NaN_3_ and incubated at 30°C for 2 hours, nutating, to inactivate a portion of the cells prior to compound addition. After this pre-treatment, the supernatant was removed and the biofilms were treated with 1:20 TSB or 20-80 µM JG-1, M4, or vehicle (DMSO) in 6% NaN_3_. The treated biofilms were incubated for 2 or 4 hours at 30°C, nutating. After incubation, the change in biofilm mass was quantified via the crystal violet assay.

An additional plate was treated the same as above and incubated in the plate reader at 30°C. RLUs were measured every 15 minutes for the duration of the experiment to monitor changes in metabolic activity.

### Rate of compound activity

Biofilms of *E. cloacae* were grown for 24 hours as described above, prior to treatment with 2.5-80 µM JG-1, M4, or vehicle (DMSO) for 1–24 hours. After treatment, biofilms were washed with H_2_O prior to fixation and staining with crystal violet as previously described (Sandala and Gunn, 2021). The change in biofilm mass was measured via crystal violet staining and used to compare rate of activity of each compound.

### RNAseq

Biofilms of *S.* Typhimurium and *E. cloacae* were grown in 96-well plates as described above. The supernatant of each well was removed and replaced with an equal volume of 10 µM (*S.* Typhimurium) or 80 µM (*E. cloacae*) JG-1, M4, or DMSO and re-incubated for 2 hours (*S.* Typhimurium) or 5 hours (*E. cloacae*). After incubation, the supernatant was removed and replaced with an equal volume of phosphate buffered saline (PBS). Plates were then scraped and/or sonicated for 10 minutes to suspend the biofilm cells. The cells were pelleted at 5000 rpm for 20 minutes at 4°C and the cell pellet frozen at -80°C for at least 30 minutes prior to RNA extraction.

Planktonic cells of *S. aureus* were grown overnight at 37°C in a rolling drum, prior to being normalized to an optical density at 600 nm (OD_600_) of 0.8 and diluted 1:1000 in 80 µM M4 or DMSO in TSB with 2.5% dextrose (TSBG). Compound treated cells were incubated for 2 hours at 37°C prior to being pelleted as described above, and frozen at -80°C for at least 30 minutes prior to RNA extraction.

RNA was isolated using the hot phenol method. In brief, frozen cells were resuspended in 2M sodium acetate (AE) buffer, 20% sodium dodecyl sulfate (SDS) (w/v), and phenol. The suspension was incubated in a 65°C water bath for 10 minutes and then immediately placed on ice. The cell debris was pelleted and the lysate treated with chloroform to separate the genomic material from the protein fraction. Any remaining protein was removed with isopropanol and cold ethanol washes until a sufficiently pure RNA pellet was obtained. The pellet was resuspended in RNase-free H_2_O and stored at -80°C until analysis.

*S.* Typhimurium library prep and sequencing was performed on the Illumina platform. Quality control and adapter trimming was performed with bcl2fastq (bcl2fastq Conversion Software). Read mapping was performed with HISAT2 (Kim et al., 2019). Read quantification was performed using Subread’s featureCounts (Liao et al., 2014) functionality. Differential gene expression was qualitied by deseq2. Pathway analysis was performed using ShinyGo 0.81 and iDEP 2.01 (Ge et al., 2018; Ge et al., 2020).

### Invasion of HeLa cells

Prior to invasion of *S.* Typhimurium, HeLa cells (gift from the Wang lab, Nationwide Children’s Hospital) were grown in 12-well tissue culture plates (Corning) with Dulbecco’s Modified Eagle Medium (DMEM, Fischer Scientific) with 10% Fetal Bovine Serum (Gibco) in 5% CO_2_ at 37°C until confluency was achieved. A single colony of *S.* Typhimurium ATCC 14028 (JSG210) from an LB agar plate was used to grow an overnight culture in TSB at 37°C. The following day, the overnight culture was diluted 1:33 in TSB or TSB supplemented with 100 µM of either JG-1, M4, or DMSO. The diluted cultures were grown statically at 37°C for 4 hours. After 4 hours, the bacterial cells from the 1:33 diluted cultures were pelleted and washed twice with TSB. The optical density of each culture was read with 1xPBS and normalized to OD_600_ = 0.8. HeLa cells were infected in quadruplicate with 1 mL of normalized cell cultures in DMEM at a multiplicity of infection (MOI) of 100. Infections were performed for 45 minutes in 5% CO_2_ at 37°C. Following infection, wells were gently washed once with 1 mL of 1x PBS and treated with 1 mL of 100 µg/mL gentamicin (Corning) in DMEM for 30 minutes in 5% CO_2_ at 37°C. The wells were then washed again with 1 mL of 1x PBS to remove any remaining gentamicin. 200 µL of 0.25% Triton X-100 (Fischer Scientific) in 1x PBS was added to each well and scraped. The scraped cells were then serially diluted and plated on LB agar plates for bacterial enumeration. Colony forming units (CFUs) were counted the next day. All invasion experiments were performed in quadruplicate.

### Motility

Liquid cultures of WT, Δ*cheY*, and Δ*tsr* isolates of *S.* Typhimurium were grown in TSB at 37°C, overnight. Following incubation, the overnight cultures were normalized to OD_600_ of 0.8 and diluted 1:100 in 1:20 TSB or 1:20 TSB supplemented with 100 µM of JG-1, M4, or DMSO. The JG-1, M4, or DMSO treated cultures, and untreated controls were incubated statically at 37°C for four hours. After pre-treatment, 5 µL of each condition was inoculated in Sulfur, Indole, Motility (SIM [NEOGEN Cat No. 50-201-5921]) agar plates supplemented with 25 µM JG-1, M4, or DMSO, or plain SIM agar. Agar plates were incubated overnight at 37°C. The diameter of the growth was measured at its widest point using calipers (Fisherbrand™ Cat. No. S90187B), to the nearest one-tenth of a millimeter (mm). Changes in motility were evaluated by comparing the diameter of growth between treatment groups.

### Target binding assays

#### Pull down

JG-1-Biotin and M4-Biotin probes were solubilized to 100 mM in DMSO and stored at -20°C. Chemical structures (**Supplementary Figure 1**) and details on synthesis can be found in Chemistry Experimental Methods. All reagents used for chemical synthesis were purchased from commercially available sources (VWR U.S., Fisher Scientific U.S., Ambeed, or Sigma Aldrich U.S.) and used without further purification. Flash chromatography was performed using 60 A° mesh standard grade silica gel from Sorbetch. Biofilms of *S.* Typhimurium and *E. cloacae* were treated with 2.5-320 µM of the probes, non-biotinylated compounds, or vehicle control (DMSO), and the change in biofilm mass was evaluated by the crystal violet assay. *S. aureus* cells were incubated in TSBG with 2.5-160 µM M4-Biotin, M4, or vehicle control (DMSO) in the SpectraMax M3 maintained at 37°C. Absorbance at OD_600_ was measured every hour for 16 hours to detect changes in cell growth.

A modified protocol from the Pierce™ Pull-Down Biotinylated Protein: Protein Interaction Kit (Thermo) was used to identify JG-1 and M4 binding proteins from *S.* Typhimurium, *E. cloacae*, and *S. aureus* lysates. Briefly, *S.* Typhimurium, *E. cloacae*, and *S. aureus* were grown in biofilm-inducing conditions for 24 hours, and cells were then collected and lysed via ultrasonic sonication. The cell debris was pelleted and the lysate was collected and stored at -20°C. Total protein concentration was quantified using the Pierce™ BCA Protein Assay Kit (Cat No. 23225) according to manufacturer protocols. The protein concentrations of each lysate are 1616.4 µg/mL (*S.* Typhimurium), 242.9 µg/mL (*E. cloacae*), and 2735.6 µg/mL (*S. aureus*).

The probes were diluted to 100 µM in 2.5 mL of each respective lysate (excluding JG-1-biotin for *S. aureus*) and incubated overnight at 4°C on a rocker to allow for compound-protein binding. Spin columns (30 µm frit) were loaded with 200 µM of NeutrAvidin^®^ UltraLink^®^ Resin slurry and washed with BupH™ Tris-Buffered Saline (TBS) prior to use. After incubation, the lysate + probe solutions were loaded onto individual columns and incubated for 4 hours at room temperature, nutating. Columns were then centrifuged at 1250 x g for 1 minute and the flow-through was collected; referred to as “(B) Lysate Flow-Through.” Columns were then washed once with TBS and the wash fraction collected; referred to as “(C) Wash.” The columns were incubated with 250 µL of Elution buffer (Pierce™, pH 2.8) for 5 minutes prior to centrifuging at 1250 x g for 1 minute to elute remaining proteins. The neutralization buffer (20 µM) was added to the collection prior to collecting the eluents. The elution step was performed twice and each sample referred to as “(D) Elution 1” and “(E) Elution 2”, respectively. Elution 1 and 2 for each respective sample and probe were combined (with 50 µL reserved for gel electrophoresis) and concentrated with a Pierce™ Protein Concentrator (3K molecular weight cutoff) to a final volume of ≈50 µL, representing a 40x concentration.

All collected column fractions and the concentrated eluents were loaded onto 4-20% or 12% SDS-PAGE and stained with SYPRO Ruby for imaging and mass spectrometry analysis. The bands of interest were excised from the gel, digested, and analyzed via nano-scale liquid chromatography-mass spectrometry-mass spectrometry (LC-MS/MS by MS-Bioworks, LLC. Data was searched using Mascot (Matrix Science) and each species’ respective databases (UniProt *Salmonella* 14028, UniProt *Staphylococcus aureus* USA300, UniProt *Enterobacter*13407 [concatenated forward and reverse plus common contaminants]). Data were filtered using 1% protein and peptide false discovery rate (FDR), requiring at least two unique peptides per protein.

#### Thermal proteome profiling

The thermal stability of bacterial proteins after incubation with compound was assessed to identify potential compound-protein interactions. Biofilms of *S.* Typhimurium were grown in 96-well plates for 24 hours as previously described. After incubation, the supernatant was removed and biofilms were treated with 10 µM JG-1, M4, or vehicle control (DMSO) in 1:20 TSB at 30°C for 2 hours. After incubation, the supernatant was removed and replaced with an equal volume and concentration of compound (or vehicle control) in PBS. The biofilms were removed from the wells by sonication in a water bath for 10 minutes. Biofilm cells were spun down and washed in PBS with compound (or vehicle control) prior to being spun down and resuspended in PBS with compound (or vehicle control). Whole protein samples of *E. cloacae* biofilms were treated with 80 µM JG-1, M4 or vehicle control (DMSO) in 1:20 LB for 5 hours. Aliquots of each cell suspension or protein fraction were heated to 25-90°C for 3 minutes and then cooled to 4°C for 3 minutes. Each sample was processed using S-Trap based digestion by the Ohio State University Proteomics Shared Resources Core and then analyzed via LC/MS-MS. Data was searched using Mascot (Matrix Science) and each species’ respective databases (UniProt Salmonella 14028, UniProt Enterobacter 13407). Data were filtered using 1% protein and peptide FDR and required at least two unique peptides per protein. The spectral counts of each of the peptides were compared at each temperature to identify proteins with increased stability at escalating temperatures.

### Mutant recalcitrance assay

To evaluate potential compound targets suggested by the performed assays, single and multiple gene mutants of *S.* Typhimurium and *S. aureus* were tested for recalcitrance to compound treatment. See **Supplementary Table 1** for a full description of all mutants tested. *S.* Typhimurium WT mutant biofilms were grown for 24 hours in 96-well plates as described previously. After incubation, the biofilms were treated with 10-160 µM of JG-1, M4, or vehicle control (DMSO) in 1:20 TSB and re-incubated for 24 hours. After treatment, change in biofilm mass was measured via the crystal violet assay. *S. aureus* WT and mutant cells were incubated in 96-well plates with 0.625-40 µM M4 or DMSO in TSBG and incubated at 37°C for 24 hours. After treatment, remaining planktonic cells were measured by OD_600_ and biofilm mass was measured via the crystal violet assay. Untreated WT biofilms were compared to mutant biofilms by an ordinary one-way ANOVA with Dunnett’s multiple comparisons test. The IC_50_/EC_50_ of JG-1 and/or M4 of WT and mutant isolates was calculated with GraphPad Prism 10.3.1 using a nonlinear regression (log(inhibitor) vs. normalized response – Variable slope Least squares fit). To identify any mutants with an altered response to the compound, the mutant IC_50_/EC_50_ was compared to the WT value calculated from the same batch of biological replicates with an extra sum-of-squares F test.

## Results

### Compound dispersal activity requires metabolically active bacteria

We have established that JG-1 and M4 exhibit anti-biofilm activity *in vitro* against *S.* Typhimurium, *S.* Typhi, and members of the ESKAPE pathogens (Sandala et al., 2020; Bennett et al., 2023). To further understand these compounds’ mechanism of action, we wanted to determine if it requires an active response from the bacteria or alternatively, if compound contact with the extracellular matrix alone is sufficient for dispersal. To differentiate these two mechanisms, we sought to induce reversible bacteriostasis in living bacteria within the biofilm, while keeping the extracellular matrix intact. To measure metabolic activity we used *S.* Typhimurium 14028lux, an isolate of *S.* Typhimurium previously described to have a chromosomal insertion of a *lux* operon (Bennett et al., 2024). Bioluminescence is dependent on ATP production, so biofilm relative luminescent units (RLU) were quantified as a proxy for ATP production.

**Figure 1:**
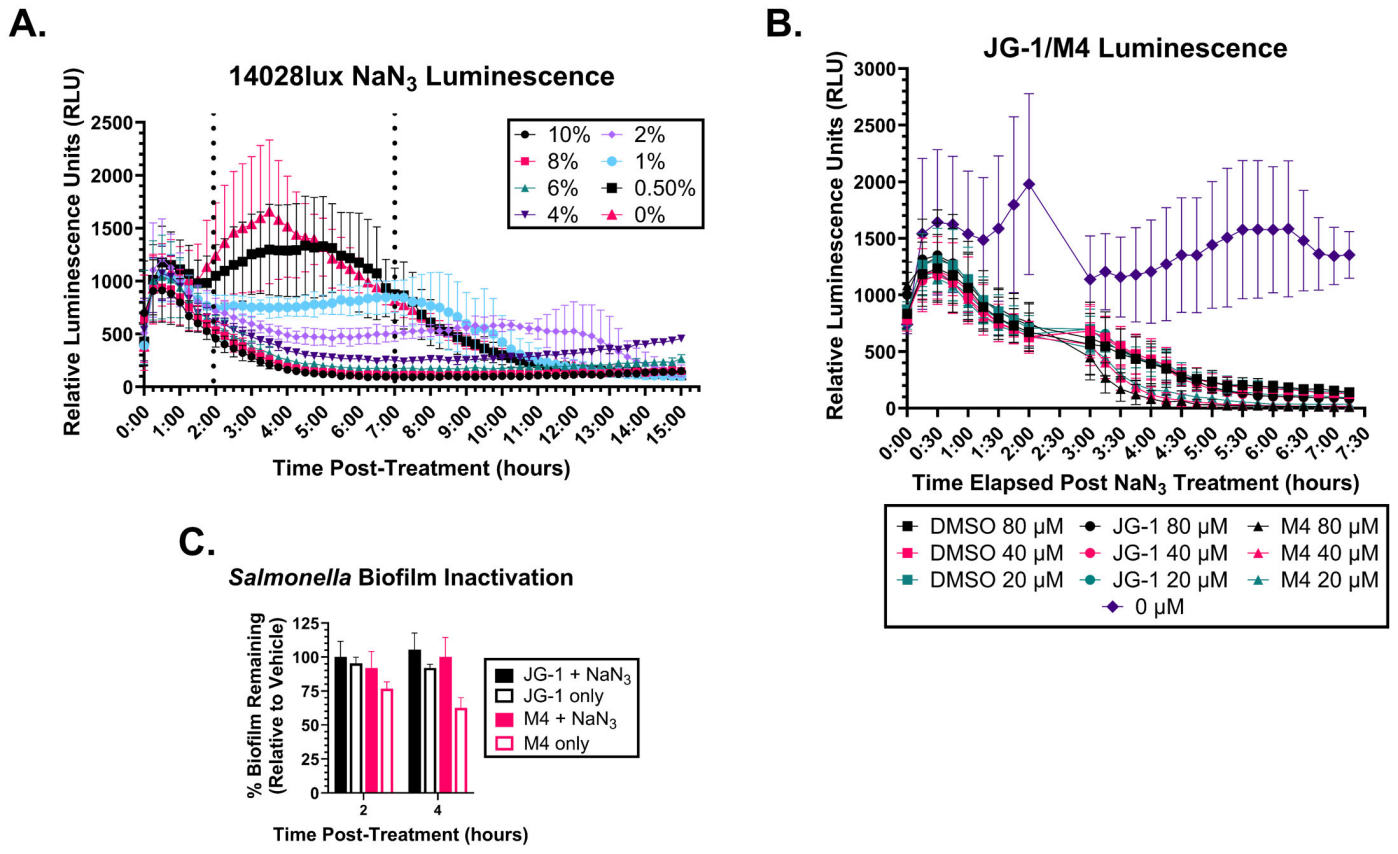
Metabolically inactive *Salmonella* biofilms do not respond to JG-1/M4 treatment. **(A)** *S.* Typhimurium 14028lux biofilms were grown in 96 well plates for 24 hours. Spent media was removed and replaced with media containing 0-10% sodium azide (NaN_3_) and re-incubated for 15 hours. Relative luminescent units (RLU) of each condition were measured ever 15 minutes to identify the minimum treatment time and [NaN3] necessary to arrest respiration. **(B)** *S.* Typhimurium 14028lux biofilms were grown in 96 well plates for 24 hours. Spent media was removed and replaced with media containing 6% NaN_3_ or fresh media (0% NaN_3_) and re-incubated for 2 hours. RLU of each condition were measured every 15 minutes. After 2 hours spent media was removed and replaced with 20-80 µM JG-1, M4, or vehicle (DMSO) in 6% NaN_3_ or fresh media (0% NaN_3_ and 0 µM compound) and re-incubated for 4.5 hours with resumption of RLU measurements every 15 minutes. **(C)** *S.* Typhimurium 14028lux biofilms were grown in 96 well plates for 24 hours. Spent media was removed and replaced with media containing 6% NaN3. After 2 hours of incubation with 6% NaN_3_, spent media was removed and replaced with either 80 µM DMSO, JG-1, or M4 in 6% NaN_3_ or 80 µм DMSO, JG-1, or M4 in fresh media. Biofilms were re-incubated for 2 and 4 hours and then washed and stained with crystal violet to measure biofilm mass. Percent biofilm remaining (relative to vehicle control) was calculated. Graphs represent average and standard deviation of data. n=3.

To induce bacteriostasis, we grew 14028lux biofilms in 96-well plates for 24 hours and then incubated them with sodium azide (NaN_3_), a bacteriostatic preservative agent that inhibits cytochrome oxidase (Lichstein and Soule, 1944). To determine a minimum time and concentration of NaN_3_ necessary to inactivate the bacteria, we treated 14028lux biofilms with varying concentrations of NaN_3_ and measured RLUs every 15 minutes for 16 hours (**Figure 1A**). We determined that incubation of a 6% NaN_3_ solution for 2 hours is the minimum concentration and time necessary to completely inactivate the bacteria. We also confirmed that the NaN_3_ inactivation can persist to at least 4 hours in the presence of compound as indicated by the low RLUs measured after compound + 6% NaN_3_ treatment (**Figure 1B**).

To determine if bacteriostasis prevents biofilm disruption, we pre-treated 14028lux biofilms with 6% NaN_3_ for 2 hours and then replaced this with media containing 80 µM JG-1, M4, or DMSO with or without 6% NaN_3_. These biofilms were incubated for either 2 or 4 hours and then washed and stained with crystal violet to quantify the percent biofilm remaining after treatment. Importantly, 6% NaN_3_ treatment leaves the biofilm structure intact as biofilms that were treated with 6% NaN_3_ alone for a total 4 or 6 hours do not show any decrease in biofilm mass (**Figure 1C**). Following pre-treatment, biofilms that were treated with JG-1 or M4 in 6% NaN_3_ had no change in biofilm mass at 2 or 4 hours (**Figure 1C**). Importantly, pre-treated biofilms that were subsequently treated with JG-1 or M4 in media without the bacteriostatic agent NaN_3_ showed a time-dependent decrease in biofilm mass by comparison to vehicle (**Figure 1C**). This indicates that metabolic inhibition with 6% NaN_3_ prevents compound induced biofilm dispersal, suggesting that the compounds’ mechanism of action requires metabolically active bacteria able to interact with and respond to the compounds.

### Treatment of bacteria with JG-1 or M4 is associated with altered transcription

Given that the compounds’ antibiofilm activity requires metabolically active cells, we predict that bacterial interaction with the compound would cause transcriptional changes that involve pathways specific to the compounds’ target. We hypothesized that the response to compound would be shared between *Salmonella* and the other members of the ESKAPE group that are susceptible to compound dispersion. To determine changes in transcription associated with compound treatment, we treated *S.* Typhimurium (WT) and *E. cloacae* biofilms with JG-1, M4, or DMSO and planktonic *S. aureus* cells treated with M4 (bactericidal effect) or DMSO, for the minimum time required to elicit a dispersion response. We have previously reported that *S. aureus* responds to M4 two hours post treatment (Bennett et al., 2023). Based on the response time indicated in **Figure 1C**, we estimate that JG-1 and M4 also require two hours of incubation in order to initiate dispersal. For *E. cloacae* biofilms with JG-1, M4, or DMSO, we determined that the first consistent decrease in biofilm mass occurs at 5 hours for both JG-1 and M4 (**Supplementary Figures 2A, B**), though we observed some rebound with M4 between hours three and four.

**Figure 2:**
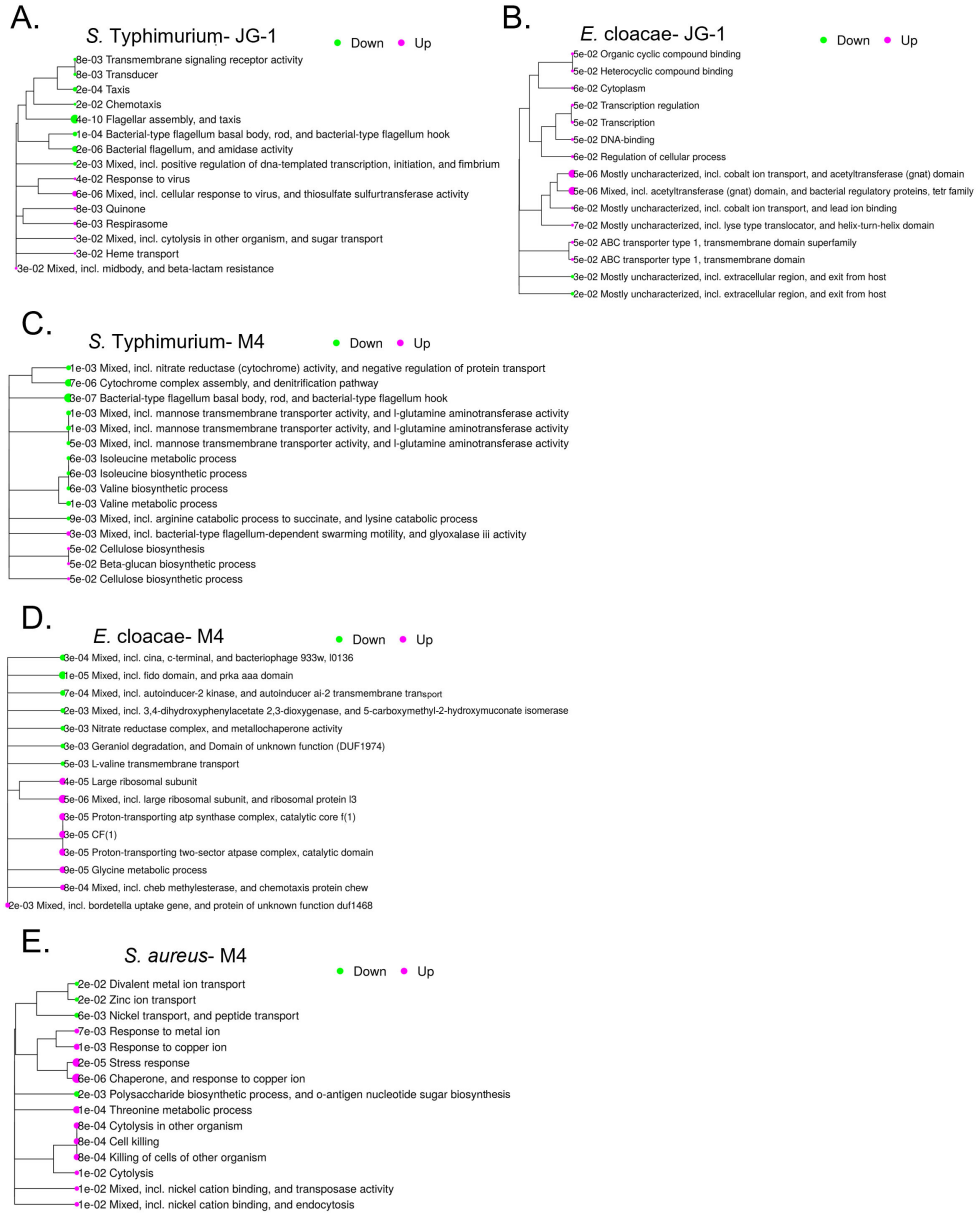
Transcriptional were changes associated with treatment with JG-1 and/or M4. Biofilms of *S.* Typhimurium and *E. cloacae* were grown in 96-well plates for 24 hours. Following incubation, biofilms of *S.* Typhimurium 14028 were treated with 10 µM JG-1, M4, or vehicle control for 2 hours. Biofilms of *E. cloacae* were treated with 80 µM JG-1, M4, or vehicle control for 5 hours. Post-treatment, planktonic cells washed away and biofilm cells collected. Planktonic cells of *S. aureus* were treated with 80 μM M4 or vehicle control for 2 hours. All treated cells were lysed and RNA collected for further analysis. Gene expression of JG-1/M4 treated cells was compared to vehicle treated cells. Data represents 3-5 biological replicates. The top 15 pathways enriched after JG-1 treatment for **(A)** *S.* Typhimurium and **(B)** *E. cloacae* are shown in relation to each other with adjusted p-values. The top 15 pathways enriched after M4 treatment for **(C)** *S.* Typhimurium and **(D)** *E. cloacae* and **(E)** *S. aureus* are shown in relation to each other with adjusted p-values. The size and color of bubbles are relative to their positive (magenta) or negative (green) normalized enrichment score Pathway performed with iDEP 2.01.

For all tested strains with compound, we unexpectedly identified hundreds of differentially expressed genes (**Supplementary Tables 2**-**4**). Therefore, pathway analysis was performed to narrow our search. The top 15 most enriched pathways for *S.* Typhimurium treated with JG-1 include a down regulation of chemotaxis, flagella, and fimbriae assembly, and upregulation of antimicrobial and phage responses (**Figure 2A**). As we expected by their different structures, the top 15 most enriched pathways for *S.* Typhimurium treated with M4 mostly differ from those of JG-1. M4 treatment results in downregulation of processes involved in respiration, amino acid metabolism, and production of the flagellar motor proteins, and upregulation of motility and extracellular polymeric substances (EPS) (**Figure 2C**). *E. cloacae* biofilms treated with JG-1 down regulate processes involved in organic compound binding, metal ion transport, and transcription broadly, while upregulating processes that are poorly understood, but predicted to be involved in escape from host stressors (**Figure 2B**). Similar to *S.* Typhimurium, *E. cloacae* biofilms treated with M4 present a very different transcriptional profile than JG-1 treated biofilms, characterized by downregulation of pathways involved in quorum sensing and membrane transport, and upregulation of pathways involved in chemotaxis, ribosome production, and protein transport (**Figure 2D**). Given the bactericidal effect of M4 against *S. aureus*, we expected to observe enrichment of stress response pathways, but were surprised to find upregulation of pathways involving cytolysis and metal ion transport (**Figure 2E**). While the pathway analysis greatly improved our understanding of how the bacteria respond to compounds, the diversity in responses suggests that their mechanism of action may be complex and involve multiple processes.

### JG-1/M4-induced transcriptional changes correlate with altered invasive and motility capacity

**Figure 3:**
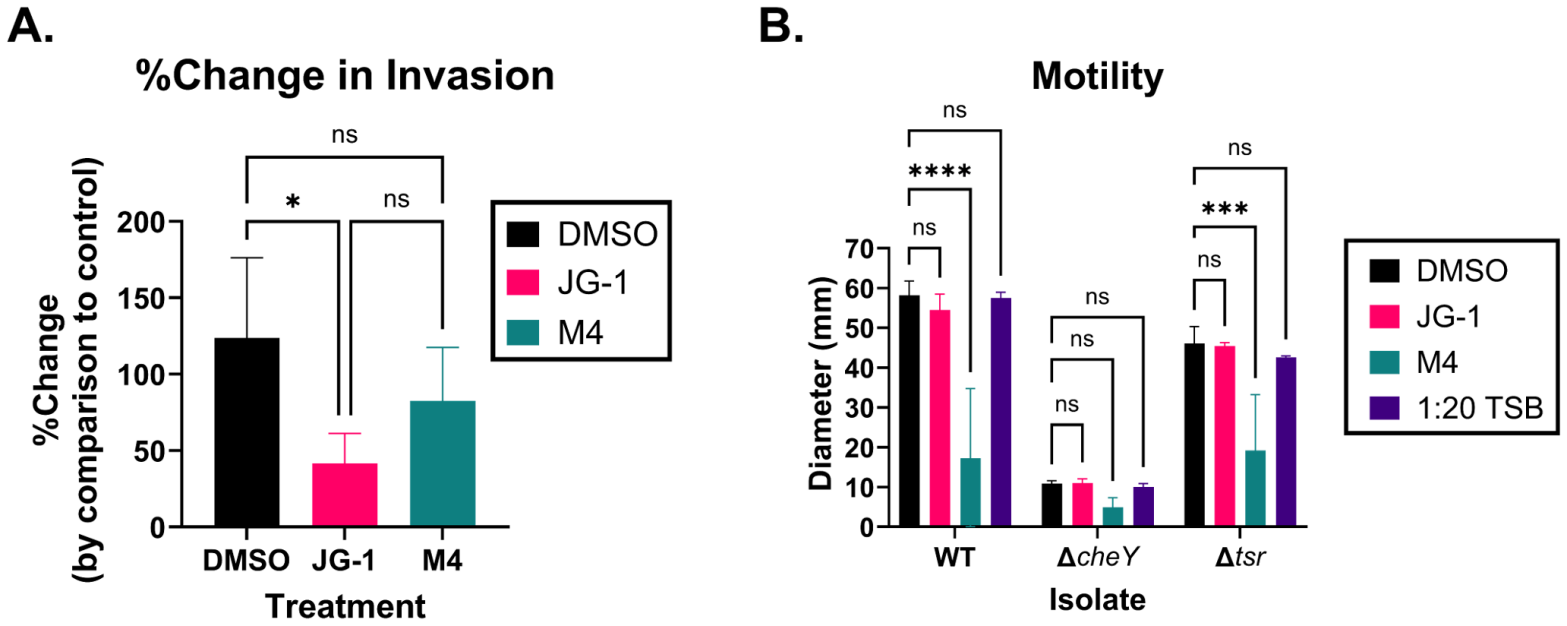
JG-1 and M4 treatment results in reduced invasion and motility in *S.* Typhimurium, respectively. **(A)** Wild-type (WT) *S.* Typhimurium planktonic cells were pre-treated with 100 µM JG-1, M4, or vehicle control (DMSO) for 4 hours at 37°C, statically. Untreated controls were incubated in media for 4 hours at 37°C, statically. HeLa cells were infected with the pre-treated bacterium, or untreated controls at a multiplicity of infection (MOI) of 100 for 45 minutes in 5% CO_2_ at 37°C. Extracellular bacteria were killed with gentamicin prior to lysing the HeLa cells and enumerating intracellular bacteria. The percentage of intracellular bacteria present in each treated group was compared to the untreated bacteria to determine the percent change (%Change) in invasion. Data represents the average and, standard deviation of %Change in invasion. Groups are compared via a one-way ANOVA with Tukey’s multiple comparisons test. n=4. **(B)** WT *S. Typhimurium*, Δ*cheY*, and Δ*tsr* mutant planktonic cells were either pre-treated with 100 µM JG-1, M4, or DMSO in 1:20 TSB, or incubated in 1:20 TSB without compound for 4 hours at 37°C, statically. 5 µL of JG-1, M4, or DMSO pre-treated *S.* Typhimurium were inoculated in Sulfur, Indole, Motility (SIM) agar plates containing 25 µM JG-1, M4, or DMSO, respectively. 5 µL of untreated control (1:20 TSB) bacteria was inoculated in plain SIM agar plates. Agar plates were incubated overnight at 37°C. The diameter of the growth on each plate was measured in millimeters (mm). Motility is represented by the average and standard deviation of the diameter of growth. The motility of the JG-1, M4, and untreated control isolates were compared to their respective DMSO treated cells via a two-way ANOVA with Dunnett’s multiple comparisons test. n=3. ns= not significant; *p<0.05; ***p<0.001; ****p<0.0001.

The results of the RNAseq and pathway analysis indicate that JG-1 and M4 induce transcriptional changes related to motility and invasion in *S.* Typhimurium. Specifically, M4 treatment results in upregulation of genes related to invasion and the SPI-1 type 3-secretion system (T3SS-1) (**Supplementary Table 2**). It is unclear whether these changes are specific to facilitating biofilm dispersal activity, or represent a phenotypic change independent of dispersal. In order to validate the RNAseq and investigate the suggested relationship between invasion and dispersal, we compared the invasion capacity of *S.* Typhimurium treated with compound or vehicle control. We infected HeLa cells with untreated WT *S.* Typhimurium or WT *S.* Typhimurium pre-treated with JG-1, M4, or vehicle. The percent of invading cells for the JG-1, M4 and DMSO treated groups were normalized to the percent invasion of the untreated bacteria to measure change in invasion induced by compound or DMSO pre-treatment. There was no significant difference in invasion between DMSO and M4 treated groups (**Figure 3A**). Surprisingly, we observed a significant reduction of invasion by JG-1 treated *S.* Typhimurium in comparison to DMSO treated bacteria (**Figure 3A**). This was unexpected because we did not observe any transcriptional changes obviously related to invasion in JG-1 treated *S.* Typhimurium.

JG-1 treatment resulted in a significant reduction of genes related to flagella, fimbriae, and chemotaxis (**Supplementary Table 2**), which are involved in adherence to and motility towards the intestinal epithelium *in vivo*, independent of the T3SS-1 (Rivera-Chávez et al., 2016). We considered that the decrease in invasion might be a byproduct of loss of motility due to the downregulation of motility-associated genes, and so we evaluated the motility of *S.* Typhimurium following JG-1/M4 treatment. We pre-treated WT *S.* Typhimurium and single gene deletion mutants of Δ*cheY* and Δ*tsr* with JG-1, M4, and DMSO in 1:20 TSB, or untreated 1:20 TSB, and measured their ability to swarm in SIM agar supplemented with the respective compounds/vehicle. Mutants lacking CheY express flagella but are non-motile, and as we expected, all treatment groups did not move from the inoculation point (**Figure 3B**). Tsr enables motility towards the intestinal epithelium, and so mutants lacking Tsr are motile and express flagella, but have an impaired invasion capacity (Rivera-Chávez et al., 2016). The Δ*tsr* mutant, as expected, was motile spreading between 40–50 mm from the inoculation point (**Figure 3B**). Importantly, DMSO treatment did not significantly alter the growth diameter in either the WT *S.* Typhimurium or the Δ*tsr* mutant, confirming vehicle alone does not alter motility (**Figure 3B**). Neither the WT nor Δ*tsr* isolates demonstrated a change in motility in response to JG-1, by comparison to vehicle (**Figure 3B**), suggesting that JG-1 does not impair motility despite the transcriptional changes observed in the RNAseq (**Supplementary Table 2**). Based on a lack of transcriptional changes observed in the RNAseq, we did not expect any changes in motility following M4 treatment; however, we observed a significant reduction in motility in the M4 treated WT and Δ*tsr* isolates, with growth diameters similar to the non-motile Δ*cheY* isolate (**Figure 3B**). The results of the invasion and motility assays together, support that JG-1 inhibits invasion, independent of motility and that M4 dramatically inhibits motility while leaving invasive capacity intact.

### Proteome profiling reveals diverse potential compound-protein interactors

**Figure 4:**
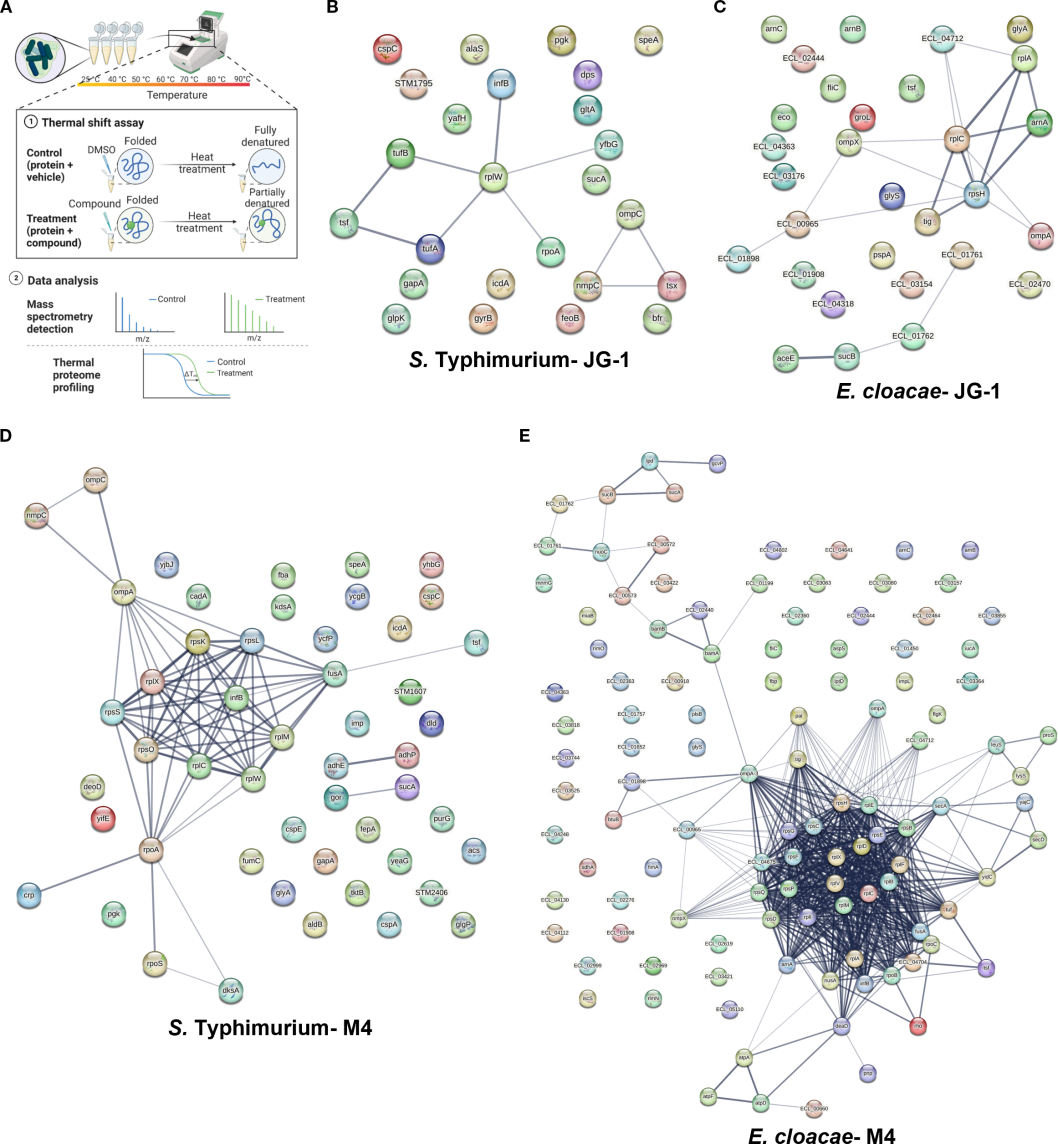
Physical subnetwork of proteins with altered melting curves identified with thermal proteome profiling. **(A)** Biofilms of *S.* Typhimurium and total protein of *E. cloacae* were incubated with 80 µM JG-1, M4, or vehicle (DMSO) and then aliquots of treated biofilm cells or protein were heated to 25-90°C, expecting that proteins bound to JG-1/M4 are stabilized, thereby increasing their melting temperature. Bacterial proteins were isolated and compared by LC/MS-MS and the results compared to the peptide database specific to the bacteria of interest (Created with BioRender.com). Criteria for positive identification included meeting a >99% protein and peptide threshold with a minimum of 2 unique peptides. Positives shifts in melting temperatures were determined by proteins with the difference in spectral counts between treated cells vs. control ≥10 in four or more ascending temperatures. The results of *S.* Typhimurium biofilms treated with **(B)** JG-1 or **(D)** M4 and *E. cloacae* proteins treated with **(C)** JG-1 and **(E)** M4 are represented in protein-protein association networks representing the predicted physical subnetwork as mined from publically available text, experiments, and databases using the STRING Database v. 12.0. An expanded table of results can be found in **Supplementary Table 5**. Proteins predicted to exist in a physical complex are connected by lines with a medium confidence (0.4). The line thickness indicates the strength of supporting data in the literature. Data represents 3 technical replicates.

To identify potential compound binding targets, we profiled the proteome of treated biofilms with a thermal shift assay. With thermal proteome profiling (TPP), proteins that are stabilized by binding to the compounds will be more resistant to heat denaturation and therefore persist at higher temperatures (**Figure 4A**). Thus, we treated *S.* Typhimurium and *E. cloacae* biofilms with compound or vehicle for 2 and 5 hours, respectively, and then heated aliquots of intact cells (*S.* Typhimurium) or whole cell purified proteins (*E. cloacae*) to temperatures between 25-90°C, thereby progressively denaturing the proteins as the temperature rises. From the resulting proteome profile, we identified >2500 individual proteins in both species and were able to identify dozens of proteins whose stability was altered in comparison to vehicle (**Supplementary Table 5**).

To identify protein-protein interactions that may be promoted by compound treatment, we wanted to determine if any of the proteins detected were predicted to interact *in situ*. Using STRING, we compared the proteins specific to each species tested that were identified in our TPP to those associations reported in the literature. We enriched for connections with significant positive predictive value of forming physical complexes *in situ* and generated interaction maps based on the results (**Figures 4B–E**; **Supplementary Table 5**). For both *S.* Typhimurium and *E. cloacae* treated with JG-1, we identified strong associations between the ribosome associated proteins and translation factors. In addition, there were strong associations with outer membrane proteins (OMP) (**Figures 4B, C**). Owing in part to the much larger list of proteins found in the TPP for *S.* Typhimurium and *E. cloacae* treated with M4, there were more numerous and complex predicted interactions than those of JG-1. Of these, there were strong associations between ribosomal structural proteins, OMPs, and membrane-associated dehydrogenases (**Figures 4D, E**). This assay presents a detailed view of how compound treatment changes global protein interactions, in addition to suggesting potential compound-protein interactors.

### JG-1 and M4 selectively bind bacterial proteins associated with biofilm formation

Performing an additional assay to examine compound-target interactions, we aimed to “fish” for these proteins by performing a pull-down assay with the compounds as “bait.” To do so, the compounds were modified to contain a biotin handle (**Supplementary Figure 1**) at a predicted neutral site. However, upon testing the activity of a biotin-modified compound (or a “pre-biotinylated” linker compound at the same site), this addition resulted in decreased activity in comparison to the native compound (**Supplementary Figure 3**). Suspecting that this reduction of activity is due to a reduced ability to penetrate cells, lysates of biofilm or planktonic cells were used to increase the chance of interactions between the biotinylated compound and their target protein(s). The resulting proteins from this pull-down assay were separated using gel electrophoresis and compared to samples incubated with vehicle only (**Supplementary Figure 4**). Upon identification of unique bands, the proteins were identified using mass spectrometry.

We identified multiple proteins suspected to bind to the biotinylated compounds (**Supplementary Table 6**). From two unique gel bands, we identified 44 total proteins in *S. aureus* cell lysates incubated with biotinylated-M4. From 7 and 8 unique gel bands (respectively), for *S.* Typhimurium and *E. cloacae* incubated with biotinylated JG-1 and M4, we identified 30 (with biotinylated-JG-1) and 15 (with biotinylated-M4) *S.* Typhimurium proteins, and 11 (with biotinylated-JG-1) and 31 (with biotinylated-M4) *E. cloacae* proteins. Because the protein targets of the two compounds are likely different, we chose to focus on proteins that were unique to each condition, resulting in 15 unique to JG-1 and 10 unique to M4 in *S.* Typhimurium, and 10 unique to JG-1 and 25 unique to M4 in *E. cloacae*. We hypothesize that *S.* Typhimurium and *E. cloacae* share the same or closely related JG-1/M4 target protein(s), and consistent with this, there were multiple orthologous proteins (>90% nucleotide similarity) identified as binding to the same compounds, including OmpA, GapA, and OmpF for JG-1 and AtpA for M4.

### Deletion of single or multiple genes results in altered response to compounds

The proteins identified in previous experiments are promising potential targets. To identify proteins that are essential to the compounds’ antibiofilm activity, we utilized single gene deletion libraries of *S.* Typhimurium and *S. aureus* to screen for resistance, or recalcitrance to JG-1 and/or M4 (a significant increase in the mutant strain’s IC_50_ or EC_50_ compared to the WT counterparts). Additionally, we assumed that because these compounds inhibit/disperse biofilm formation, any mutant lacking a true target should demonstrate a deficiency in biofilm formation.

In total, we tested 160 single and multiple gene deletion mutants of *S.* Typhimurium and 21 single gene deletion mutants of *S. aureus* (**Supplementary Table 7**). These mutants included genes encoding proteins identified in the TPP and pull-down, genes identified in the RNAseq, and genes included in the top enriched pathways. We evaluated biofilm-forming ability by measuring the mass of mutant versus WT biofilms grown in plain media for either 24 (*S. aureus*) or 48 hours (*S.* Typhimurium). In *S.* Typhimurium, we identified 41 individual mutations that resulted in significantly less biofilm mass (and 5 individual mutations that resulted in significantly more biofilm mass than WT) (**Supplementary Table 7**). In *S. aureus*, we identified four mutations that resulted in significantly less biofilm mass than WT: Δ*codY*, Δ*atl*, Δ*ebpS*, and Δ*SAUSA300_2097* (**Supplementary Table 7**).

*S.* Typhimurium mutants were tested for resistance to JG-1 and M4 dispersal. We first examined genes with altered transcription as evidenced by the RNAseq and pathway analysis (**Figure 2**; **Supplementary Table 2**). We tested mutants of auto-inducer-2 (AI-2) genes *ego* (*lsrA*), *luxS, tqsA, ydeV*, and *ydeW*, and the *N*-acyl homoserine lactone (AHL) sensor *sdiA*. Of those genes, only the Δ*sdiA* mutant was recalcitrant to M4 activity (**Table 1**). We also wanted to evaluate the role of cyclic-di-GMP signaling in compound-triggered dispersion. We tested multiple mutants of genes involved in this process, including *yaiC* (*adrA*), *typA*, *yciR*, and *yegE.* The Δ*yaiC* mutant was more sensitive to JG-1 treatment than WT but had no baseline biofilm deficit (**Table 1**). Curiously, the Δ*yegE* mutant was recalcitrant to M4 activity, but produced more biofilm than WT (**Table 1**). We observed a similar increase in biofilm formation in Δ*ptsN*, Δ*rseC*, and Δ*yhbH* mutants (**Supplementary Table 7**). The TPP results for both *S.* Typhimurium and *E. cloacae* treated with M4 suggested an increase in ribosomal protein stability (**Figure 4**); therefore, we examined *S.* Typhimurium genes related to ribosome stability and stalling, including: *lepA, prfC, relB, relE, rimI, rplI, rsd, STM14_1162, tig, yajC, yciR*, and *yhbH.* Deletion of LepA results in recalcitrance to both JG-1 and M4 (**Table 1**). Deletion of RimI results in increased susceptibility to JG-1 and M4, while deletion of RelE results in recalcitrance to JG-1 and deletion of RplI, STM14_1162, YciR, or YhbH results in recalcitrance to M4 (**Table 1**).

**Table 1:** *Salmonella* gene deletions resulting in a significant change in JG-1/M4 EC_50_.

JSG	Target gene(s)	Gene symbol	Mutation polarity	Δ Biofilm mass	n=	JG-1 logEC_50_	WT vs. mutant JG-1 logEC_50_ p-value	M4 logEC_50_	WT vs. mutant M4 logEC_50_ p-value	↓ EC_50_	↑ EC_50_
5321	STM14_0014	*dnaJ*	Nonpolar	↓	2	-2.967	0.3782	Unstable	0.0001	N	Y- M4
5533	STM14_0077	*carA*	Nonpolar	↓	3	-5.415	0.6186	Unstable	0.0466	N	Y- M4
5534	STM14_0077	*carA*	Polar	↓	3	-2.231	0.981	Unstable	0.0144	N	Y- M4
5289	STM14_0161	*yacA*	Nonpolar	N	2	-3.479	0.5696	-3.894	0.0204	N	Y- M4
5143	STM14_0220	*dksA*	Polar	N	3	-1.136	0.0164	-3.482	0.9415	N	Y- JG-1
5488	STM14_0247	*pfs*	Nonpolar	N	3	-2.59	0.7251	-3.752	0.0051	N	Y- M4
5441	STM14_0265	*rseP*		↓	3	-59.86	<0.0001	-4.025	0.2222	N	Y- JG-1
4658	STM14_0455	*yaiC*	Nonpolar	N	3	-2.161	0.5104	-3.867	0.0716	Y- JG-1	N
5514	STM14_0622	*arcC*	Nonpolar	↓	3	-2.791	0.6763	-3.764	0.0021	N	Y- M4
5166	STM14_0862	*cydB*	Polar	N	2	-0.5389	0.0017	-4.453	0.8957	Y- JG-1	N
5303	STM14_1162		Nonpolar	N	2	-2.718	0.4496	-3.946	0.0034	N	Y- M4
5197	STM14_1214	*ompA*	Nonpolar	↓	6	-4.686	0.0282	-3.257	0.0062	Y- JG-1	Y- M4
4856	STM14_1304	*csgF*	Polar	↓	2	1.466	0.0168	-4.35	0.288	N	Y- JG-1
4570	STM14_1310	*csgA*	Nonpolar	↓	2	Unstable	CC	-9.588	0.0357	N	Y- M4
5547	STM14_1577	*gdhA*	Nonpolar	N	2	-3.867	0.0711	-4.003	0.0042	N	Y- JG-1
5524	STM14_1903	*narU*	Nonpolar	N	3	Unstable	CC	-3.927	0.0058	N	Y- M4
5471	STM14_2058	*yciR*	Nonpolar	N	3	-11.25	0.0008	-13.33	0.0008	N	Y- M4
5532	STM14_2134	*narK*	Polar	N	3	-3.08	0.6915	-3.701	0.0092	N	Y- M4
5557	STM14_2138	*narX*	Nonpolar	N	2	-3.082	0.7408	-218120	CC	Y- M4	N
5133	STM14_2168	*cydB* homolog	Nonpolar	N	2	-2.978	0.0392	-4.192	0.8115	N	Y- JG-1
5540	STM14_2170	*gluD* homolog	Nonpolar	N	2	Unstable	0.2412	-3.722	0.0222	N	Y- M4
4851	STM14_2337	*motB*	Nonpolar	N	2	-3.357	0.7365	0.9759	0.0399	N	Y- M4
5477	STM14_2368	*sdiA*	Nonpolar	N	3	-7.6	0.0027	17.36	0.3647	Y- JG-1 ≤20 µM	Y- JG-1 ≥40 µM
5489	STM14_2368- STM14_2416		Polar	↓	3	-2.597	0.8594	-4.074	0.021	N	Y- M4
4855	STM14_2373	*fliZ*	Polar	N	2	-3.496	0.901	-3.494	0.0306	N	Y- M4
3733	STM14_2378	*fliC*	Nonpolar	N	2	-2.984	0.0366	-3.779	0.3885	N	Y- JG-1
5162	STM14_2603	*wcaG*	Nonpolar	N	3	-2.847	0.3604	Unstable	0.0048	N	Y- M4
4744	STM14_2620	*yegE*	Nonpolar	↑	3	-3.163	0.0784	-3.884	<0.0001	N	Y- M4
5542	STM14_2775	*narP*	Nonpolar	N	2	-3.107	0.5837	-3.711	0.0002	N	Y- M4
5187	STM14_2797	*ompC*	Polar	↓	5	-3.623	0.041	-3.802	0.0573	N	Y- JG-1
5574	STM14_2797	*ompC*	Polar	N	3	-2.166	0.0343	-4.336	0.3145	N	Y- JG-1
5549	STM14_2874	*yfbQ*	Nonpolar	N	2	Unstable	CC	-0.3867	<0.0001	Y- M4	N
5543	STM14_3042	*narQ*	Nonpolar	N	2	-2.732	0.9883	-3.401	0.0004	N	Y- M4
5302	STM14_3135	*hmpA*	Polar	N	2	-1.933	0.0499	Unstable	0.1055	N	Y- JG-1
5290	STM14_3163	*lepA*	Nonpolar	N	2	-3.671	0.0207	-4.174	0.0267	N	Y- JG-1/M4
5384	STM14_3231	*rseC*	Nonpolar	N	3	-1.912	0.2051	-3.856	0.02	N	Y- M4
5385	STM14_3231	*rseC*	Polar	↑	3	-0.38	0.0046	-3.64	<0.0001	N	Y- JG-1/M4
5439	STM14_3231	*rseC*		N	3	-2.889	0.2387	-3.648	0.0009	Y- M4 ≤20 µM	Y- M4 ≥40 µM
5366	STM14_3233	*rseA*	Nonpolar	N	3	-3.581	0.2935	-4.148	0.0345	N	Y- M4
5367	STM14_3233	*rseA*	Polar	↓	3	-3.537	0.4843	-3.533	0.0067	N	Y- M4
5320	STM14_3234	*rpoE*	Nonpolar	↓	2	-10.09	<0.0001	-22.02	0.0933	Y- M4	N
4071	STM14_3720	*speB*	Nonpolar	N	2	-3.227	0.0012	-5.632	0.2532	N	Y- JG-1
5530	STM14_3973	*argG*	Nonpolar	↓	3	-2.942	0.3813	-4.089	0.0236	N	Y- M4
5297	STM14_3976	*secG*	Nonpolar	N	2	-2.737	0.4152	-3.33	0.0009	N	Y- M4
5173	STM14_4008	*rpoN*	Nonpolar	N	2	Unstable	0.037	Unstable	0.0001	N	Y- JG-1/M4
5372	STM14_4009	*yhbH*	Nonpolar	↑	3	-2.814	0.5703	Unstable	0.0052	N	Y- M4
5373	STM14_4009	*yhbH*	Polar	N	3	-3.282	0.8297	Unstable	0.0013	N	Y- M4
5382	STM14_4010	*ptsN*	Nonpolar	↑	3	-1.321	0.0002	-3.559	<0.0001	N	Y- JG-1/M4
5383	STM14_4010	*ptsN*	Polar	↑	3	-3.019	0.7173	-3.629	<0.0001	N	Y- M4
5388	STM14_4012	*ptsO*	Nonpolar	N	3	-2.187	0.2517	Unstable	0.0114	Y- M4 ≤20 µM	Y- M4 ≥40 µM
5440	STM14_4041	*degS*		↓	3	112541	0.2495	-5.422	<0.0001	N	Y- M4
5315	STM14_4173	*crp*	Nonpolar	N	2	-7011187	CC	-3.944	0.0032	N	Y- M4
5307	STM14_4461	*secB*	Nonpolar	↓	5	-2.481	0.0275	-4.491	<0.0001	Y- JG-1/M4	N
5210	STM14_4507	*spoT-Δctd*	Polar	N	3	-8.607	0.2952	-3.662	0.0138	N	Y- M4
5390	STM14_4659		Polar	N	3	-3.507	0.3914	-3.241	0.0225	Y- M4 ≤20 µM	Y- M4 ≥40 µM
5380	STM14_4660	*atpC*	Nonpolar	↓	3	-5.05	0.7157	Unstable	0.0347	N	Y- M4
5381	STM14_4660	*atpC*	Polar	↓	3	Unstable	CC	Unstable	0.0412	N	Y- M4
5378	STM14_4662	*atpG*	Nonpolar	↓	3	Unstable	CC	Unstable	0.0411	N	Y- M4
5379	STM14_4662	*atpG*	Polar	↓	3	Unstable	CC	Unstable	0.0481	N	Y- M4
5132	STM14_4711	*gppA*	Nonpolar	N	2	-20.49	0.0944	-3.775	0.0002	N	Y- M4
3718	STM14_4713	*trxA*	Nonpolar	N	2	-2.32	0.0049	-7.484	0.3584	N	Y- JG-1
5573	STM14_4713	*trxA*	Nonpolar	N	3	2.887	0.0107	-4.028	0.0082	N	Y- JG-1/M4
5537	STM14_4820	*glnA*	Polar	↓	3	-7.156	<0.0001	-3.967	0.429	Y- JG-1 ≤40 µM	Y- JG-1 ≥80 µM
5484	STM14_4899	*ego*	Polar	N	3	-2.926	0.7276	-3.925	0.0159	N	Y- M4
5528	STM14_4955	*argE*	Nonpolar	N	3	-1.634	0.9992	-3.927	0.0253	N	Y- M4
5538	STM14_4956	*argC*	Nonpolar	N	3	Unstable	CC	-4.004	0.0121	N	Y- M4
5539	STM14_4956	*argC*	Polar	↓	3	-3.596	0.283	-3.876	0.0088	N	Y- M4
5487	STM14_5242	*hfq*	Polar	↓	3	-3.668	0.4782	-5.031	0.0201	N	Y- M4
5296	STM14_5278	*rplI*	Nonpolar	N	2	-2.828	0.4374	-3.36	0.0057	N	Y- M4
5136	STM14_5341	*relE*	Polar	N	2	-2.792	0.0115	-4.229	0.3989	N	Y- JG-1
5522	STM14_5358	*argF* homolog	Nonpolar	N	3	-2.83	0.6803	-3.821	0.0086	N	Y- M4
5322	STM14_5476	*rimI*	Polar	↓	5	-3.422	0.0929	-4.495	0.0041	Y- JG-1/M4	N
5375	STM14_5477	*yjjG*	Nonpolar	N	3	Unstable	CC	-3.009	<0.0001	N	Y- M4
5444		*degS rseP*		↓	3	-2.204	0.9424	-192598	0.0084	N	Y- M4
5578		*ompC ompF*	Polar/Nonpolar	N	3	-1.075	0.0249	-4.392	0.3336	N	Y- JG-1
5577		*ompC trxA*	Polar/Nonpolar	N	3	-1.683	0.0314	-4.456	0.0925	N	Y- JG-1
5353		*secB rimI*	Nonpolar/Polar	↓	3	-3.178	0.0407	-5.131	<0.0001	Y- JG-1/M4	N

Biofilms of *Salmonella* mutants were grown in 96-well plates prior to treatment with JG-1, M4, or vehicle in order to identify changes in biofilm mass and dispersal activity (EC_50_) by comparison to WT. All mutants with a change in dispersal activity are listed with details on their background, change in baseline biofilm mass, and JG-1/M4 logEC_50_ with p-values. Change in biofilm mass was calculated by comparing untreated mutant biofilm mass to WT biofilm mass using a one-way ANOVA with Dunnett’s multiple comparisons test. All changes represent significant (p<0.05) increases or decreases in biofilm mass. Change in logEC_50_ was calculated by comparing mutant EC_50_ to WT with an extra sum-of-squares F test (α=0.05). n=2-6; N=no; Y=yes; Δ= change; ↑= increase; ↓= decrease; CC=cannot calculate.

In total, we identified 21 *S.* Typhimurium mutants that exhibit an increased EC_50_ in response to JG-1 treatment, and 53 that have an increased EC_50_ in response to M4 treatment (**Table 1**). In total, 17/53 mutants were both intrinsically deficient in biofilm mass and had an increased EC_50_ to only M4. Additionally, 4/21 of the mutations were both intrinsically deficient in biofilm mass and had an increased EC_50_ to only JG-1 (Δ*rseP*, Δ*csgF*, Δ*ompC*, and Δ*glnA*). Notably, OmpC and OmpF were identified with JG-1 in the pull-down, and OmpC was associated with increased stability at higher temperatures in the TPP. However, OmpF did not have a biofilm deficit or change in EC_50_ to either compound, in comparison to WT. Interestingly, we also identified 14 mutants that had an unexpected decrease in EC_50_ (increased susceptibility) in response to JG-1 and/or M4 treatment. Notable among these genes is *ompA*, whose loss conferred increased sensitivity to JG-1 and increased resistance to M4. OmpA was identified as binding to both JG-1 and M4 in the pull-down (**Supplementary Table 6**).

Given the interest in *ompA* and *ompC* as targets, we back-transduced these mutations from the library derived isolates, JSG 5197 and JSG 5187, respectively, into our WT strain (**Supplementary Table 1**). This allows us to ensure that the phenotype is a result of the gene deletion and not an unlinked mutation. We compared the new Δ*ompA* and Δ*ompC* mutants, JSG 5575 and JSG 5574, respectively, to WT and identified that JSG 5575 (Δ*ompC*) retains its parental JG-1 resistance phenotype, while JSG 5574 (Δ*ompA*) does not recapitulate the increased sensitivity to JG-1 or recalcitrance to M4 seen in the parent strain. (**Supplementary Table 7**).

Given the evidence supporting OmpC as an important JG-1 target *in vitro*, we determined if loss of OmpC affected the *in vivo* response to JG-1. To evaluate this, we used a murine model of typhoidal chronic carriage previously used to demonstrate JG-1 efficacy *in vivo* after infection with WT *S.* Typhimurium (Sandala et al., 2020; Woolard et al., 2022). 129X1/SvJ mice were fed a lithogenic diet to induce gallstone formation prior to being infected with *S.* Typhimurium Δ*ompC* (JSG 5574). Five days post-infection, mice were assigned to three treatment groups and given daily ip. injections of either 1 mg/kg Cipro (Cipro only), 1 mg/kg Cipro in 5% DMSO (DMSO+Cipro), or 10 mg/kg JG-1 and 1 mg/kg Cipro in 5% DMSO (JG-1+Cipro), in a volume of 100 µL sterile PBS for 10 days. Post-treatment, the mice were euthanized and the bacterial load in the gallbladder, liver, spleen, cecum, and whole blood were compared between groups. There was a significant decrease in bacterial load in the gallbladder of the JG-1+Cipro group by comparison to the Cipro only and DMSO+Cipro groups (**Supplementary Figure 5**). Therefore, we concluded that OmpC is dispensable for JG-1 efficacy *in vivo* despite the promising importance *in vitro*.

*S. aureus* mutants were evaluated for both M4 antibiofilm activity at low concentrations, and M4 bactericidal activity at higher concentrations. Of the 21 *S. aureus* mutants tested, 16 had an altered response to M4 treatment in comparison to WT. Specifically, Δ*manA*, Δ*sbi*, Δ*deoC*, Δ*SAUSA300_2097*, and Δ*hchA* mutants were more resistant to M4 killing than WT (**Table 2**). Additionally, Δ*codY*, Δ*ahpC*, and Δ*hchA* mutants were resistant to M4 antibiofilm activity, as evidenced by their significant increase in IC_50_ in comparison to WT (**Table 2**). Of note, only Δ*codY* was both resistant to M4 antibiofilm activity and deficient in biofilm formation at baseline. Thus, we propose that while bactericidal activity is facilitated by binding to multiple proteins, M4’s antibiofilm mechanism of action in *S. aureus* is facilitated by interacting with CodY.

**Table 2:** *S. aureus* gene deletions resulting in a significant change in M4 IC_50_.

JSG	Target gene	Gene symbol	Δ Biofilm mass	n=	Cells			Biofilm		
					logIC_50_	WT vs. mutant M4 logIC_50_ p-value	Δ IC_50_	logIC_50_	WT vs. mutant M4 logIC_50_ p-value	Δ IC_50_
5316	SAUSA300_1148	*codY*	↓	3	-4.75	0.2941	N	-6.307	0.0086	↑
5318	SAUSA300_2479	*cidA*	N	3	-4.732	0.0587	N	-5.019	0.1257	↓
5326	SAUSA300_2540	*fda*	N	3	-4.848	0.1363	N	-5.622	0.0002	↓
5327	SAUSA300_0594	*adh*	N	3	-4.927	0.1189	N	-5.317	0.0118	↓
5329	SAUSA300_2518		N	3	-4.923	0.1299	N	-5.153	0.0207	↓
5331	SAUSA300_1902		N	3	-4.921	0.1587	N	-5.166	0.0031	↓
5332	SAUSA300_0380	*ahpC*	N	3	-4.941	0.4914	N	-5.053	0.0009	↑
5333	SAUSA300_2573	*isaB*	N	3	-4.851	0.0867	N	-5.743	0.0002	↓
5334	SAUSA300_2096	*manA*	N	3	-4.738	0.0002	↑	-5.221	0.3561	N
5335	SAUSA300_2364	*sbi*	N	3	-4.865	0.0144	↑	-4.99	0.5723	N
5336	SAUSA300_2486	*clpL*	N	3	-4.926	0.9538	N	-5.232	0.0196	↓
5338	SAUSA300_0140	*deoC*	N	3	-4.828	0.0104	↑	-5.03	0.5644	N
5339	SAUSA300_1370	*ebpS*	↓	3	-4.798	0.2994	N	-5.239	0.0101	↓
5341	SAUSA300_2097		↓	3	-4.871	0.0434	↑	-5.252	0.0003	↓
5343	SAUSA300_0235	*ldh1*	N	3	-4.911	0.2287	N	-5.17	0.0088	↓
5344	SAUSA300_0536	*hchA*	N	3	-4.708	0.0009	↑	-4.83	0.0378	↑
5345	SAUSA300_0736	*hpf*	N	3	-4.934	0.5563	N	-5.204	0.0151	↓

*S. aureus* mutants were incubated with M4, or vehicle in 96-well plates in order to identify changes in biofilm mass and M4 cell and/or biofilm inhibitory activity (IC_50_) by comparison to WT *S. aureus*. All mutants with a change in response to compound activity are listed with details on their background, change in baseline biofilm mass and M4 logIC_50_ with p-values. Change in biofilm mass was calculated by comparing untreated mutant biofilm mass to WT biofilm mass using a one-way ANOVA with Dunnett’s multiple comparisons test. All changes represent significant (p<0.05) increases or decreases in biofilm mass. Change in logIC_50_ was calculated by comparing mutant IC_50_ to WT with an extra sum-of-squares F test (α=0.05). n=3, N= no; Y= yes; Δ= change; ↑= increase; ↓= decrease.

## Discussion

In this study, we expand our understanding of the mechanism of action and cellular interactions of JG-1 and M4, antibiofilm molecules with broad-spectrum activity against multiple bacterial pathogens including *Salmonella*, *Enterobacter* spp., and *S. aureus*. We first aimed to determine if the compounds act on the biofilm structure or live bacteria within the biofilm. While some antibiofilm agents function by causing a phenotypic change in the bacteria (Janssens et al., 2008; Wexselblatt et al., 2012; Hassanov et al., 2018; Lyu et al., 2024), other agents act by targeting the structural integrity of the biofilm matrix (Goodman et al., 2011; Matilla-Cuenca et al., 2021; Jurcisek et al., 2022). By inactivating the bacteria with NaN_3_ without disrupting the biofilm matrix, we were able pinpoint that the compound’s mechanism of action depends on live bacteria within the matrix, not the matrix alone.

This prompted us to ask how the biofilm bacteria respond to compound. We used RNAseq to measure changes in transcription in response to compounds. We limited this search to the transcriptional changes associated with early interaction with compound and found this to be a highly dynamic event, with dozens of differentially expressed genes. *S.* Typhimurium biofilm response to JG-1 is characterized, in part, by downregulation of pathways associated with fimbriae and flagella. Flagella and curli fimbriae are important components of the *Salmonella* biofilm (Jonas et al., 2007; Adcox et al., 2016; El Hag et al., 2017), so it is reasonable that bacteria preparing to disperse would reduce production of these adhesive agents. Flagella and fimbriae also contribute to motility and attachment to host-cells. We propose that the reduction in these appendages may contribute to the decrease in invasion observed in JG-1 treated *S.* Typhimurium. Less clear is the role of reduction of chemotaxis and flagella-mediated motility; however, this may reflect reduction of aggregative chemotaxis rather than general motility. This is supported by the results of the motility assay, which suggests JG-1 has no effect on motility in SIM agar. The pathway analysis of *E. cloacae* biofilm response to JG-1 is characterized by upregulation of genes involved in organic compound binding and transcription regulation. We suspect that because JG-1 is an organic compound, this may be a generic response to similar compounds. We hypothesize that the upregulation of transcription may be reactivation of sessile *E. cloacae* cells as they prepare to disperse from the biofilm. This suggests that JG-1 may potentiate increased metabolic activity within biofilms, a state that is also associated with increased antibiotic susceptibility (Das et al., 2019).

In response to M4, *Salmonella* upregulates pathways associated with flagellar motility and genes specific to the T3SS-1 and type six secretion system (T6SS). Both the T3SS-1 and T6SS have been shown to contribute to invasion of host cells *in vivo* (Bao et al., 2020). Initially these results suggested to us that M4 induces a transition to a motile and pro-invasive state. This was contradicted by the results of the motility assay, which demonstrated M4 causes a dramatic reduction in motility. Interestingly, while M4 causes a decrease in motility, it does not compromise invasion capacity, as there was no difference in invasion of HeLa cells between the M4 and vehicle treated groups. Evidence in the literature suggests that flagellar motility and the T3SS-1 independently contribute to invasion because mutants deficient in either system are still able to invade murine Peyer’s patches, although to a lesser degree than WT *S.* Typhimurium (Rivera-Chávez et al., 2016). Our results support that flagellar motility is not essential for invasion as the less motile M4-treated *S.* Typhimurium invade HeLa cells comparably to vehicle treated bacteria. It is likely that upregulation of invasion-associated genes such as T3SS-1 genes *spaM* and *spvC*, T6SS genes *tssG* and *impA* (*STM14_0313*), and colonic acid biosynthesis genes *wcaF and wcaE* ensure invasion capabilities without significantly increasing this capacity. While colonic acid is dispensable for *Salmonella* biofilm formation on plastic or gallstones, evidence suggests that *S.* Typhimurium uses colonic acid to adhere to mammalian epithelial cells and plant cells (Salmonella in domestic animals; Prouty et al., 2002; Ledeboer and Jones, 2005).

*Salmonella* form a flagellar T3SS (fT3SS) that allows them to form and rotate flagella under diverse circumstances, using either proton motive force across the membrane or an ATPase ring complex (Minamino et al., 2016; Biquet-Bisquert et al., 2021; Minamino et al., 2021; Minamino et al., 2022). This system can also be used to generate membrane potentials sufficient to communicate across biofilms, independent of motility (Masi et al., 2015; Prindle et al., 2015; Biquet-Bisquert et al., 2021). Evidence in *Bacillus subtilis* shows that increased electrical signaling promotes biofilm formation by attracting nearby *P. aeruginosa*, and that this mechanism may be generic across bacterial species (Humphries et al., 2017; Biquet-Bisquert et al., 2021). Therefore, we propose that the decrease in transcription associated with cytochrome activity, transmembrane transport, and the flagelllar motor observed in the M4 *S.* Typhimurium pathway analysis may be representative of decreased intra-biofilm electrical signaling, rather than a transition to a motile state, as evidenced by the loss of motility following M4 exposure. A similar phenotype is evident in the pathway analysis of *E. cloacae* biofilms treated by M4, with decreases in inter-biofilm signaling molecules and transmembrane transport, and increases in chemotaxis pathways.

Thermal proteome profiling revealed a very large number of proteins with increased stability following compound treatment. There were few commonalities in structure or sequence between the positive hits, thus it is unlikely that JG-1 or M4 are truly stabilizing every protein identified. Some of these proteins may be stabilized by binding to compound; however, stabilization can also occur from increased binding to other proteins. Moreover, given the diversity of transcriptional changes, it would not be unreasonable for treatment to radically change how the proteome interacts. This hypothesis is supported by the physical subnetwork predictions, as many of the hits are predicted to exist in physical complexes with one another. For example, M4 drives increased stability of ribosomal proteins in both *S.* Typhimurium and *Enterobacter* spp. While this could be interpreted as the compound stabilizing these proteins, we argue that this is reflective of changes in ribosome metabolism. The bacterial ribosome can act as a small molecule sensor and respond by arresting assembly to control gene expression (Bhattacharya et al., 2024). A protective response to antibiotic or nutritional stress common across bacteria is to post-translationally modify structural ribosomal proteins or ribosome assembly factors to increase stability of ribosomal subunits, either to arrest transcription to conserve resources or increase translational fidelity (Connolly and Culver, 2009; Siddiqui, 2021; Maksimova et al., 2022; Ciolli Mattioli et al., 2023; Ni et al., 2023; Chan and Groisman, 2024). We observe increased stability of ribosomal proteins and their predicted translation factors as a response to both compounds in both *S.* Typhimurium and *Enterobacter* spp. and we propose that many of these may represent increased native-native protein interactions, driven by upstream stress caused by the compounds. Supporting this theory, we also observed increased transcription of ribosomal proteins and translation factors. All that considered, many proteins did not have predicted physical interactions, partially due to gaps in the literature, particularly in the case of *E. cloacae*, these proteins may actually be evidence of potential compound-protein interactions.

A strength of the pull-down approach is that it is highly specific for compound-protein binding. As expected, we identified fewer proteins in the pulldown than the TPP, owing to its greater specificity. Following up on potential target proteins, we tested *S.* Typhimurium single deletion mutants of genes identified in the pull down and TPP, as well as differentially expressed pathways, for changes in JG-1 and/or M4 activity. We assumed that the deletion of any genes essential to the compound mechanism of action should reduce the biofilm-forming capacity of the resulting mutant by comparison to WT bacteria. Many of the mutants tested are well established to be poor biofilm-producers in *Salmonella*, including those required for producing fimbriae (Barnhart and Chapman, 2006; Jonas et al., 2007; Adcox et al., 2016; Ahmad et al., 2017; El Hag et al., 2017; Mares, 2017; Rehman et al., 2019; González et al., 2024; Lyu et al., 2024) (CsgA, CsgD, CsgF, PefA, SecB, YacA, YajC), flagella (Crawford et al., 2010b; Osterman et al., 2015; Nord et al., 2017; Horstmann et al., 2020; Wang et al., 2020) (STM14_2368-STM14_2416), stringent response (Azriel et al., 2015) (RelA, GppA) and other stress responses (Monteiro et al., 2012; Emerging and divergent roles of pyrophosphorylated nucleotides in bacterial physiology and pathogenesis, 2021; Cohen et al., 2022) (DegS DksA, Hfq, KatE, RpoE, YciE). We also identified multiple genes that are important to *in vitro S.* Typhimurium biofilm formation that were not previously reported in the literature, including *degS, rseA*, and *rseP*. Findings about the role of OmpC’s *S.* Typhimurium virulence *in vivo* vary, with some studies suggesting Δ*ompC* mutants have decreased colonization capacity during mouse infection (Role of ompR-dependent genes in Salmonella typhimurium virulence: mutants deficient in both ompC and ompF are attenuated in vivo; van der Heijden et al., 2016), while others do not find a virulence deficit in comparison to WT *S.* Typhimurium (Dorman et al., 1989; Li et al., 2021). In this study, we demonstrate that Δ*ompC* is deficient in *in vitro* biofilm formation, but still able to colonize the gallbladder during mouse infection, suggesting that OmpC is not essential for biofilm formation in the gallbladder in this gallstone-mediated chronic infection. Additionally, we did not observe an loss in JG-1+Cipro efficacy in Δ*ompC* infected mice, suggesting that while JG-1 and OmpC may interact *in vitro*, this interaction may not be sufficient *in vivo* to trigger biofilm dispersal. This was surprising due to the strong evidence of its importance to JG-1 action *in vitro.* Future studies into proteins essential to JG-1 antibiofilm action *in vivo* will be necessary to resolve this question.

Production of respiratory complexes and electron acceptors is essential to *Salmonella* virulence *in vivo* (Lopez et al., 2012; Maier et al., 2013; Kokkinias et al., 2024), but their role in biofilm formation has not been well described. In *Escherichia coli*, cytochrome *bd* production is essential for biofilm development (Beebout et al., 2019). However, in the conditions we tested, *S.* Typhimurium mutants of *cydB, STM14_2168*, *cyoA, cyoB*, and *cyoE* form biofilms as well as WT *S.* Typhimurium. Additionally, deletion of *narL*, a gene reported to be important to *S.* Typhimurium biofilm formation (Role of narL gene in the pathogenesis of Salmonella Typhimurium - PubMed), did not have an effect on biofilm formation. Moreover, none of the mutations of the nitrogen sensing nor metabolism genes we tested (*napB, napF, narK, narL, narP, narQ, narU, narX, nirB*) had any impact on biofilm production.

Our investigation of the mechanism of action of M4 against *S. aureus* focused on teasing out the mechanisms contributing to M4’s dual bactericidal and antibiofilm properties. We chose to explore the transcriptional profile of *S. aureus* cells treated with a bactericidal concentration of M4 to understand the basis for its activity outside of an antibiofilm context. The pathway analysis revealed that *S. aureus* responds to high concentrations of M4 by upregulating pathways associated with cytolysis and cell killing. Notably these pathways are induced by sensing of foreign cells and foreign cell lysis and typically are involved in toxin production and lysis of other bacteria and host cells (PANTHER: Making genome-scale phylogenetics accessible to all, 2022; Carbon and Mungall, 2024). This suggests that the bacteria are producing toxin despite no other species to target. We hypothesize that this results in a “friendly fire” event as the bacteria succumb to their own excessive lytic toxin production rather than the disruption of internal processes resulting in cell death.

Notably M4 has a divergent activity in *S. aureus*, inhibiting biofilm formation at low concentrations and killing the cells at concentrations above this threshold (Bennett et al., 2023). In the pull-down assay, the concentration used to treat the *S. aureus* cell lysate was intended to select for protein binding that results in cell death. However, it is likely to also pulldown proteins related to antibiofilm activity. We identified more proteins that bind to M4 in *S. aureus* than either of the other species tested, suggesting that M4 may bind to a more diverse array of proteins in *S. aureus*. This also suggests that the dual activity may be caused by binding to more than one protein. Of the proteins identified in the pull-down and followed up with recalcitrance testing, only the loss of CodY reduced biofilm forming capacity and conferred resistance to M4’s antibiofilm activity. CodY is a global transcriptional regulator of *Staphylococcal* biofilm and toxin production; activation of CodY enhances biofilm formation and represses virulence factor secretion by binding the promotor region of specific toxin genes and preventing transcription (Otto, 2008; Schilcher and Horswill, 2020; Patel and Rawat, 2023). However, when nutrients are scarce, CodY’s binding affinity for DNA decreases, allowing for increased transcription of toxin genes and reduced transcription of biofilm genes (Patel and Rawat, 2023). In this context, we propose that M4 elicits a similar response by preventing CodY activation and thereby inhibiting biofilm formation.

The individual deletion of *manA*, *sbi, deoC*, *SAUSA300_2097*, or *hchA* was sufficient to confer resistance to M4’s *S. aureus* bactericidal activity. Notably, deletion of *hchA* confers resistance to both antibiofilm and bactericidal activity; however, we would like to emphasize the caveat that because Δ*hchA* mutants produce similar amounts of biofilm to WT, inhibiting the action of HchA is not likely the main effector of M4’s antibiofilm activity. With this in consideration, we believe that M4 binding CodY is the major mechanism of action for its antibiofilm properties in *S. aureus.* However, Δ*codY* mutants are viable, and do not succumb to excessive toxin production, in part, because *Staphylococci* mediate damage by producing proteins involved in detoxification and repair (Hochgräfe et al., 2008; Wolf et al., 2008; Fuchs et al., 2013). Notably, multiple examples of these proteins were identified as binding M4 in the pull down, including AhpC, DnaK, HchA, Ldh1, RplY, Tsf, Tuf, and TrxB. Of these, loss of HchA confers resistance be to M4 killing. HchA detoxifies phosphorylated glycolytic intermediates under conditions of nitrosative stress (Bäsell et al., 2014). We propose that at higher concentrations, M4 binding CodY and HchA, triggers excess toxin production and impairs the cells’ response to the increased stress, respectively, ultimately leading to demise of the cell. At lower concentrations, more HchA is free to mediate stress caused by CodY inactivation, and thus the cells are able to survive, but unable to form biofilms.

In this study, we report on the complex mechanism of action of two small molecule compounds, JG-1 and M4, in multiple bacterial species. We focus our study on three pathogens, *S.* Typhimurium, *Enterobacter* spp., and *S. aureus*, as they are representative of the antibiofilm and/or bactericidal activity we have previously reported. With our multifactorial approach, we determined that JG-1 and M4 trigger biofilm dispersal not by inhibiting a single protein, but multiple protein interactions important to maintaining the biofilm state. Additionally, we identified that M4’s antibiofilm and bactericidal activity in *S. aureus* is likely facilitated by binding to CodY, preventing transcription of biofilm genes and inducing excessive toxin production. M4 also binds to other *S. aureus* proteins that mediate toxic stress, and overwhelms the cell’s compensatory mechanisms, leading to its demise. Together these results shed light on the mechanism of action of JG-1 and M4 *in vitro* in multiple species, and help inform its therapeutic capacity against chronic biofilm-mediated infections.

## Data Availability

The original contributions presented in the study are publicly available. This data can be found here: https://doi.org/10.5061/dryad.9ghx3ffwm.

## Supplementary material

Supplementary Table 1 Isolate List.

Supplementary Table 2
*Salmonella* RNAseq.

Supplementary Table 3
*Enterobacter* RNAseq.

Supplementary Table 4
*Staphylococcus* RNAseq.

Supplementary Table 5 TPP proteins.

Supplementary Table 6 PullDown proteins.

Supplementary Table 7 IC/EC_50_ Summary.

Supplementary Figure 1 Structure of original, pre-biotin, and biotinylated compounds. **(A)** JG-1 **(B)** pre-biotin JG-1 **(C)** biotinylated JG-1 **(D)** M4 **(E)** pre-biotin M4 **(F)** biotinylated M4.

Supplementary Figure 2
*Enterobacter cloacae* biofilms disperse in a time-dependent manner in response to JG-1/M4. Following growth for 24 hours in 96-well plates, spent media was removed from *E. cloacae* biofilms and replaced with media containing 2.5-80 µM JG-1, M4, or vehicle control. Biofilms were the re-incubated for 0-24 hours and following incubation washed and stained with crystal violet to measure changes in biofilm mass. Graphs represent average and standard deviation of percent biofilm remaining after treatment with **(A)** JG-1 or **(B)** M4. n= 1-7.

Supplementary Figure 3 Activity of the pre-biotin JG-1/M4 compared to the original compound. JG-1/M4 compounds were synthesized with a pre-biotin or biotin addition. **(A)** *S.* Typhimurium cells were incubated with 0.78-100 µM of M4, M4 pre-biotin, JG-1, JG-1-pre-biotin, or equal volume of vehicle control (DMSO) in 1:20 TSB. Inhibition of biofilm formation (IC_50_) was measured using the crystal violet assay to quantify changes in biofilm formed by comparison to DMSO treated cells. n=1 **(B)** *Enterobacter* spp. biofilms were grown for 24 hours in 96-well plates prior to treating the biofilms with 1.56-320 µM of M4, M4 pre-biotin, JG-1, JG-1-pre-biotin, or DMSO for 24 hours. Dispersion of biofilm mass (EC_50_) was measured using the crystal violet assay to quantify changes in biofilm remaining by comparison to DMSO treated cells. IC_50_/EC_50_ was calculated with GraphPad Prism 10.3.1 using a nonlinear regression model. n=3 **(C)** *S. aureus* planktonic cells were treated with 80 µM of M4, M4 pre-Biotin, M4 Biotin, or an equal amount of DMSO in TSBG and incubated at 37°C, statically. the OD_600_ was measured every hour for 16 hours in order to quantify cell growth in each condition. Data represents average and standard deviation of OD_600_ at each time point. n=5.

Supplementary Figure 4 Isolation of proteins identified with the pulldown assay. **(A)** Following incubation of biotinylated JG-1 and M4, or vehicle with *S.* Typhimurium, *E. cloacae*, and *S. aureus* cell lysates, the solution was filtered through a NeutrAvidin column. Unbound proteins were washed away and the remaining bound protein fraction was saved for purification and further analysis. Figure created with BioRender.com. **(B-E)** The eluted fraction from each condition was concentrated with a 3K MCO centrifugal filter and then run on 12% SDS-PAGE gels with a 250kD ladder for reference. Following electrophoresis, gels were fixed and stained with SYPRO Ruby and imaged in order to visualize protein bands. Arrows indicate unique bands in the JG-1 or M4 lanes that were excised and further analyzed. The identified proteins for each band are presented in **Supplementary Table 6**.

Supplementary Figure 5 Treatment with JG-1 and/or ciprofloxacin does not reduce gallbladder bacterial burden in mice infected with a Δ*ompC S.* Typhimurium. Male and female 6-8 week old 129X1/SvJ mice were fed a lithogenic diet for 8 weeks. Following resumption of normal diet, mice were infected via an intraperitoneal (ip.) injection with 3.3x10^3^ colony forming units (CFUs) of a *S.* Typhimurium 14028 isolate with a deletion of *ompC*. Five days post-infection the mice were treated with an ip. injection of 1 mg/kg ciprofloxacin (Cipro), 1 mg/kg Cipro in 5% DMSO (v/v), or 10 mg/kg JG-1 and 1 mg/kg Cipro in 5% DMSO (v/v), in a volume of 100 µL sterile PBS, per day, for 10 days. After day 10 of treatment, the mice were euthanized and gallbladder, liver, spleen, cecum, and blood collected for enumeration of CFUs. Graph represents average and standard deviation of CFUs from each organ. Organ CFUS are compared between treatment groups via a two-way ANOVA with Holm-Šídák’s multiple comparisons test. *p<0.05, ns, not significant.
