# Supplementary material for: Mechanisms of antibiofilm compounds JG-1 and M4 across multiple species: alterations of protein interactions essential to biofilm formation
Source: Front Cell Infect Microbiol. 2025 Sep 17;15:1631575. doi: 10.3389/fcimb.2025.1631575 (PMC12484186; doi:10.3389/fcimb.2025.1631575)
Supplement: Supplementary file 1 [file DataSheet1.pdf]

**Chemistry Experimental:** All reagents used for chemical synthesis were purchased from commercially available sources (VWR U.S., Fisher Scientific U.S., or Sigma Aldrich U.S.) and used without further purification. Flash chromatography was performed using 60 Å mesh standard grade silica gel from Sorbetch. NMR solvents were obtained from Cambridge Isotope Labs and used as is. All $^{1}H$ NMR and $^{13}C$ NMR were recorded at 25 ˚C on Bruker AVANCE III HD spectrometers (400 MHz). Chemical shifts (δ) are given in parts per million (ppm) relative to the respective NMR solvent; coupling constants (*J*) are in hertz (Hz). Abbreviations used are s, singlet; d, doublet; dd, doublet of doublets; t, triplet; m, multiplet. High-resolution mass spectrometry measurements were obtained at the Notre Dame Department of Chemistry Mass Spectrometry and Proteomics Facility. Infrared spectra were obtained on a Bruker Alpha II FTIR spectrophotometer ($\nu_{max}$ in $cm^{-1}$). UV absorbance was recorded on a Genesys 10 scanning UV/visible spectrophotometer ($\lambda_{max}$ in nm). The purities of the tested compounds were all verified to be ≥ 95% by LC-MS analysis on an Advion LC-MS 2020 with Kinetex, 2.6 mm, $C_{18}$ 50 × 2.10 mm, using 20-100% acetonitrile/water with 0.1% formic acid for either 5 or 3 minutes.

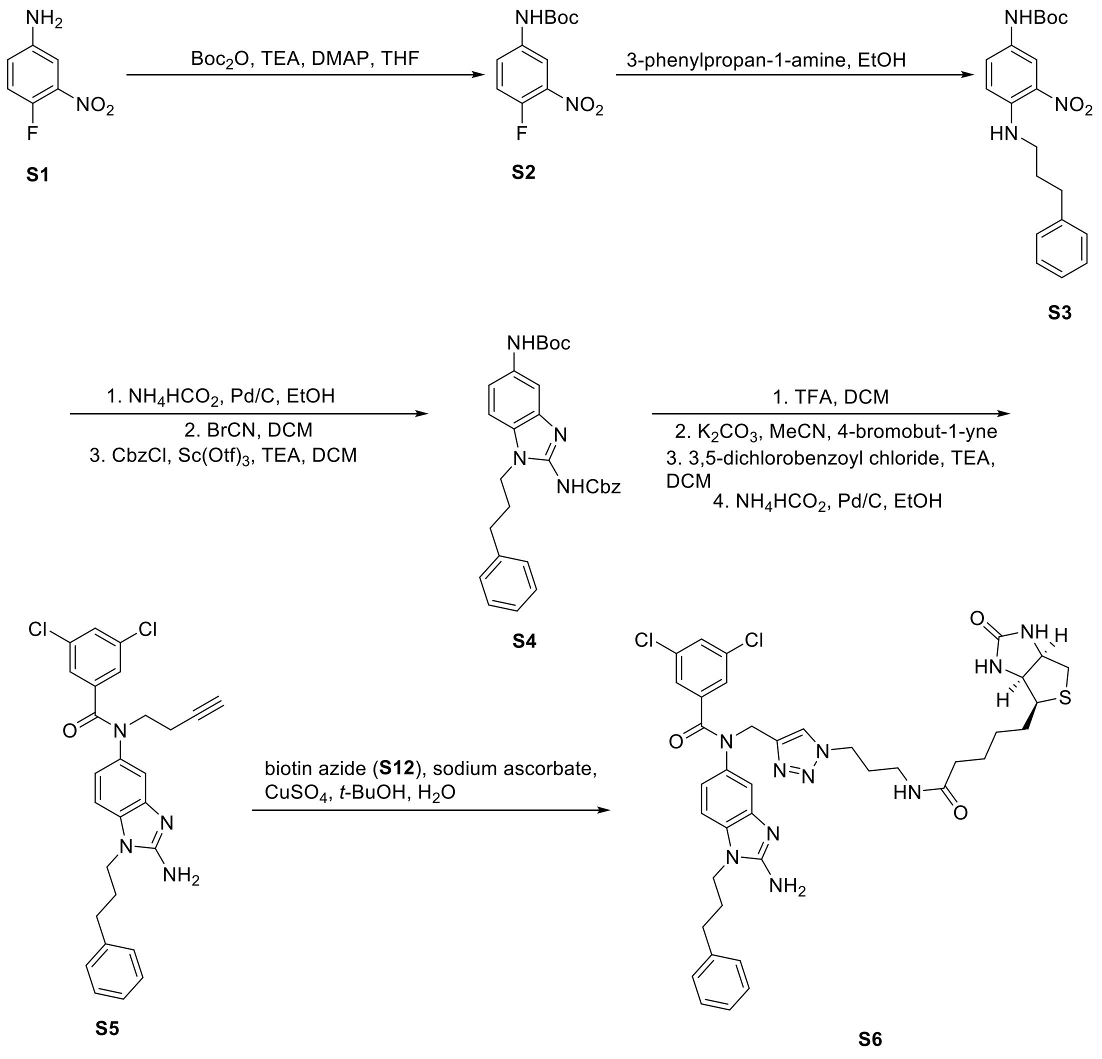


**Scheme 1.** Synthesis of biotinylated M4 (**S6**)

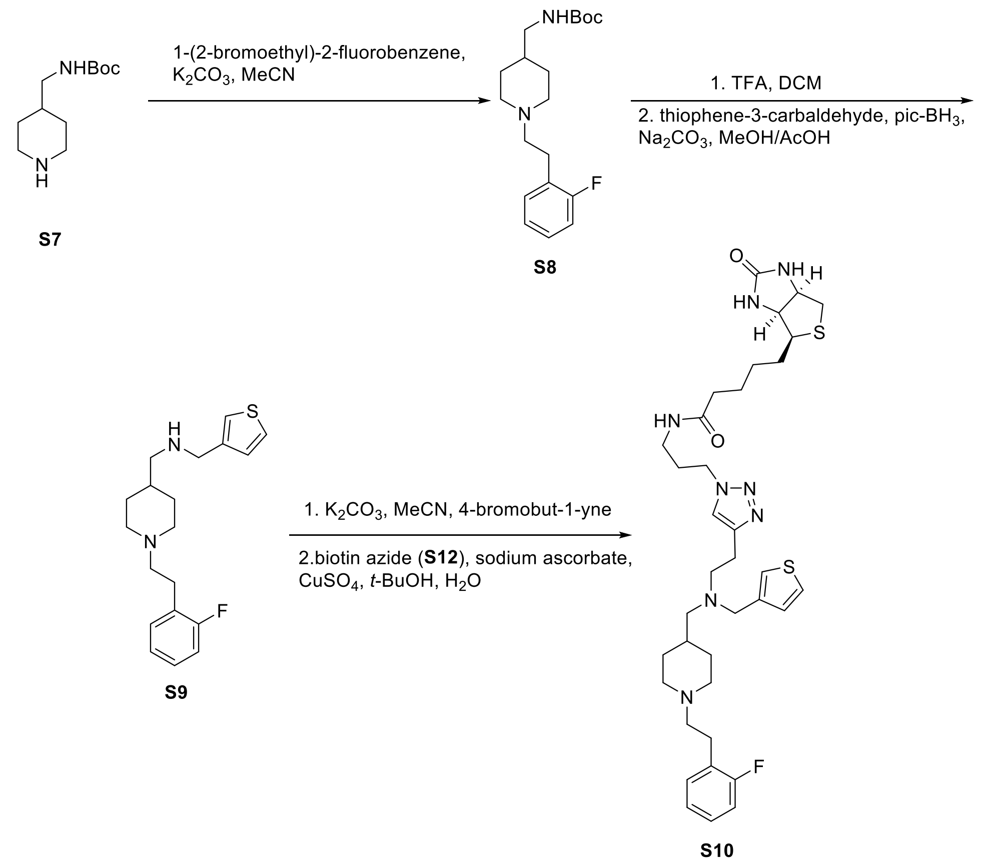


**Scheme 2.** Synthesis of biotinylated JG-1 (**S10**)

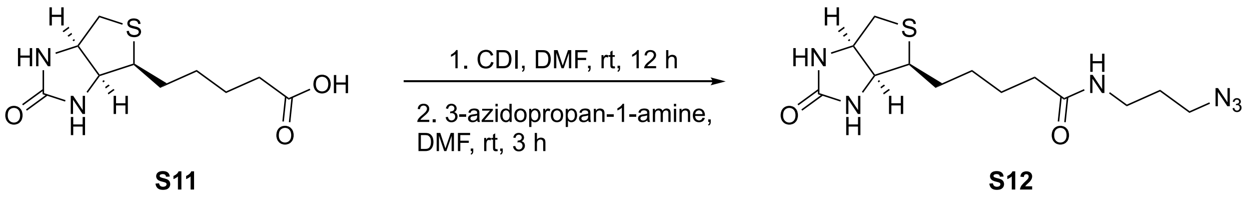


**Scheme 3.** Synthesis of biotin azide (**S12**)

## Previously Reported Compounds

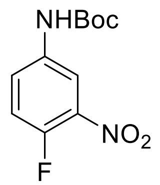

***tert*-Butyl (4-fluoro-3-nitrophenyl)carbamate (S2):** Compound was synthesized using previously reported methods.[1] Spectral data was consistent with previous reports.[1]

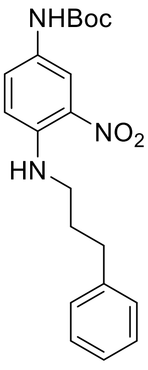


***tert*-Butyl (3-nitro-4-((3-phenylpropyl)amino)phenyl)carbamate (S3):** Compound was synthesized using previously reported methods.[1] Spectral data was consistent with previous reports.[1]

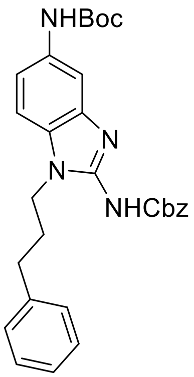


**benzyl tert-butyl (1-(3-phenylpropyl)-1H-benzo[d]imidazole-2,5-diyl)dicarbamate (S4):** Compound was synthesized using previously reported methods.[1] Spectral data was consistent with previous reports.[1]

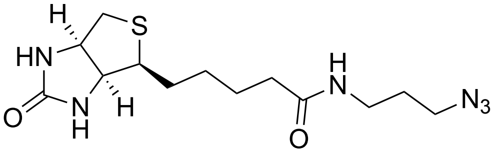


**N-(3-azidopropyl)-5-(2-oxohexahydro-1H-thieno[3,4-d]imidazol-4-yl)pentanamide (S12):** Compound was synthesized using previously reported methods.[2] Spectral data was consistent with previous reports.[2]

**Novel Compound Characterization**

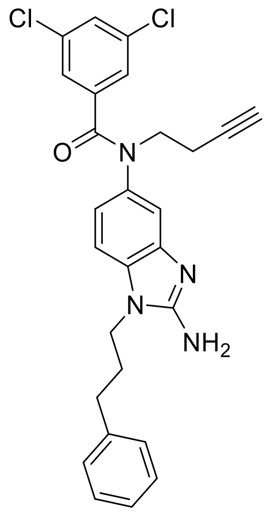

***N-(2-amino-1-(3-phenylpropyl)-1H-benzo[d]imidazol-5-yl)-N-(but-3-yn-1-yl)-3,5-dichlorobenzamide* (S5):** Compound **S4** (220 mg) was dissolved in 30% TFA/DCM (30 mL) under an inert atmosphere (Ar) at 0 °C. The reaction was allowed to stir for 1 h, and upon completion via TLC analysis (1.5 triethylamine:1.5 methanol:97.5 $CH_2Cl_2$), the solvent was then removed under reduced pressure. The crude intermediate (250 mg) was then dissolved in acetonitrile (30 mL) and dry potassium carbonate (259 mg) was added. The solution was heated to reflux. 4-bromobut-1-yne (187 mg) was dissolved in acetonitrile (5 mL) and was slowly added to the reaction over an hour via syringe pump. The reaction was then cooled to room temperature and concentrated under reduced pressure. The resulting residue dissolved in ethyl acetate (30 mL) and washed with 1x brine (30 mL). The organic layer was collected and dried over sodium sulfate. The sodium sulfate was filtered off and the resulting organic layer was condensed under reduced pressure and dissolved in anhydrous dichloromethane (20 mL) and was placed under an inert atmosphere (Ar). To the solution triethylamine (0.25 mL) and 3,5-dichlorobenzoyl chloride (204 mg) were added. The reaction was stirred at room temperature for an hour. The organic layer was then washed 1x with 1N HCl (15 mL), 1x with saturated sodium bicarbonate (30 mL), and 1x with brine (30 mL). The organic layer was then dried over sodium sulfate. The sodium sulfate was filtered off and the resulting solution was concentrated under reduced pressure and dissolved in ethanol. Pd/C (7 mg.) was added to the flask and was heated to reflux. Ammonium formate (559 mg) was then added to the refluxing solution slowly over an hour. The reaction mixture was cooled to room temperature and filtered through a pad of celite. The crude product was washed 1x with saturated sodium bicarbonate (30 mL). The organic layer was collected and then dried over sodium sulfate, the sodium sulfate removed via filtration, and the solvent was removed under reduced pressure. The crude product was purified via flash chromatography (1-5% methanol-ammonia/ dichloromethane to yield a tan solid (71 mg, 32% yield). $^1$H NMR (400 MHz, dimethyl sulfoxide-$d_6$) δ 8.92 (s, 2H), 7.53 (s, 1H), 7.49 (d, *J* = 8.54 Hz, 1H), 7.37 (s, 1H), 7.33 (s, 1H), 7.26 – 7.19 (m, 3H), 7.18 – 7.11 (m, 3H), 4.64 (s, 2H), 4.18 (t, *J* = 7.15 Hz, 2H),

3.27 (s, 1H), 2.66 – 2.58 (m, 2H), 2.00 – 1.89 (m, 2H); $^{13}$C NMR (101 MHz, dimethyl sulfoxide-$d_6$) δ 166.8, 151.0, 141.3, 139.5, 137.3, 134.1, 130.0, 129.6, 129.5, 128.7, 128.4, 127.1, 126.3, 123.8, 111.9, 111.0, 79.4, 75.9, 42.7, 32.2, 29.5; UV ($\lambda_{max}$ nm): 290 nm; IR ($\nu_{max}$ cm$^{-1}$): 3260, 2337, 1658; HRMS (ESI) calc'd for $C_{26}H_{22}Cl_2N_4O$ [M+H]$^-$: 511.0865, found: 511.0869.

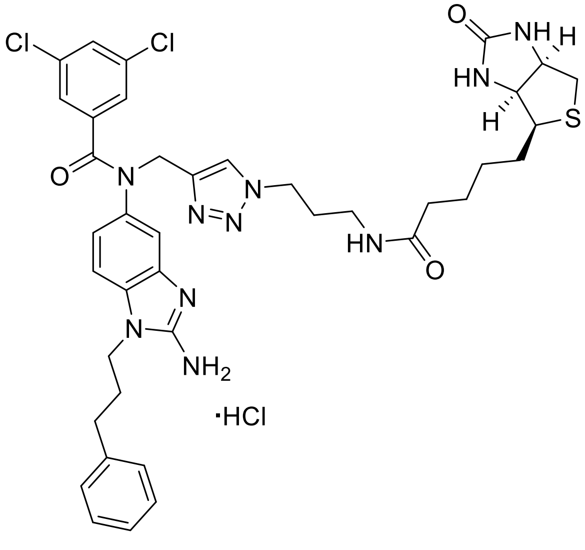


***N*-(2-Amino-1-(3-phenylpropyl)-1H-benzo[d]imidazol-5-yl)-3,5-dichloro-N-((1-(3-(5-((3aS,4S,6aR)-2-oxohexahydro-1H-thieno[3,4-d]imidazol-4-yl)pentanamido)propyl)-1H-1,2,3-triazol-4-yl)methyl)benzamide (S6):** To a round bottom flask, *N*-(2-amino-1-(3-phenylpropyl)-1H-benzo[d]imidazol-5-yl)-3,5-dichloro-*N*-(prop-2-yn-1-yl)benzamide (1 equiv, 37 mg) and 5-[(3as,4s,6ar)-2-oxo-1,3,3a,4,6,6a-hexahydrothieno[3,4-d]imidazol-4-yl]-n-(3-azidopropyl)pentanamide (1.0 equiv, 24 mg) were added to a 1: 1 mixture of water and *tert*-butanol (0.5 mL:0.5 mL). A freshly-prepared solution of sodium ascorbate in water (0.05 equiv, 0.71 mg) was added to the solution followed by the addition of a catalytic amount of copper (II) sulfate pentahydrate (0.01 equiv, 0.18 mg). The solution was then stirred at room temperature overnight and the reaction was then diluted with water, cooled over ice, and filtered. The solid was washed with cold water and dried under vacuum. The solid was then purified via flash column chromatography (5% methanol-ammonia/ dichloromethane). The product was then dissolved in methanol (5 mL) and concentrated hydrochloric acid (1 mL) was added to the solution. The solution was then concentrated under reduced pressure. To remove excess water, anyhydrous ethanol was added (3 mL) and the subsequently removed under reduced pressure. This was repeated 2 additional times and the resulting solid dried under vacuum overnight to yield a pale yellow solid (4 mg, 17 % yield). $^1$H NMR (400 MHz, dimethyl sulfoxide-$d_6$) δ 8.13 (s, 1H), 7.90 (t, *J* = 6.04 Hz, 1H), 7.60 (t, *J* = 7.35 Hz, 1H), 7.45 (s, 1H), 7.32 (s, 2H), 7.30 – 7.26 (m, 1H), 7.24 (d, *J* = 7.37 Hz, 2H), 7.17 (d, *J* = 6.94, 1H), 7.12 (d, *J* = 7.44 Hz, 2H), 6.97 (s, 2H), 6.77 – 6.69 (m, 1H), 6.44 (s, 1H), 6.37 (s, 1H), 5.30 (s, 1H), 5.02 (s, 2H), 4.36 – 4.26 (m, 3H), 4.14 – 4.09 (m, 1H), 3.97 (t, *J* = 6.20 Hz, 2H), 3.13 – 3.04 (m, 5H), 3.03 – 2.96 (m, 4H), 2.21 – 2.10 (m, 3H), 2.07 (t, *J* = 7.28 Hz, 2H), 1.95 – 1.83 (m, 5H); $^{13}$C NMR (101 MHz, dimethyl sulfoxide-$d_6$) δ 180.8, 175.5, 168.2, 163.2, 156.6, 150.4, 144.3, 139.3, 137.1, 136.6, 134.7, 130.8, 128.8, 128.5, 127.2,

127.1, 126.3, 124.2, 119.5, 78.0, 67.2, 61.4, 59.6, 58.3, 55.8, 52.4, 52.0, 47.6, 46.0, 35.7, 32.5, 30.4, 29.4, 26.2, 25.8; UV ($\lambda_{max}$ nm): 266 nm; IR ($\upsilon_{max}$ cm$^{-1}$): 2360, 2344, 2248; HRMS (ESI) calc'd for $C_{39}H_{44}Cl_2N_{10}O_3S$ [M+H]$^+$: 803.2768, found: 803.2776.

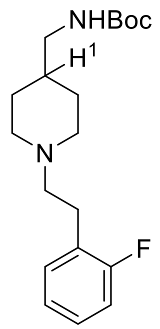


**tert-Butyl ((1-(2-fluorophenethyl)piperidin-4-yl)methyl)carbamate (S8):**
To an oven dried round bottom flask, 1-(2-bromoethyl)-2-fluorobenzene (1 equiv., 2.43 g ), anhydrous acetonitrile (57 mL), potassium carbonate (1.5 equiv., 2.35 g), and tert-butyl (piperidin-4-ylmethyl)carbamate (1.2 equiv., 2.77 g ) were added. The reaction vessel was purged with Ar and then heated to reflux with stirring under Ar overnight. The acetonitrile was removed under reduced pressure and the product was extracted with dichloromethane (50 mL). The organic solution was washed with water (1x, 40 mL) and brine (1x, 40 mL) and then was dried over sodium sulfate. The sodium sulfate was removed via filtration, the collected organic layer was concentrated under reduced vacuum and the resulting crude oil purified via flash chromatography with 2.5:2.5:95 methanol:triethylamine:dichloromethane to afford **S8** as a light brown oil (3.17 g, 83% yield). $^1$H NMR (400 MHz, methanol-$d_4$) δ 7.30 – 7.24 (m, 1H), 7.24 – 7.20 (m, 1H), 7.13-7.08 (m, 1H), 7.08-7.01 (m, 1H), 3.06 (m, 2H), 2.96 (d, *J* = 6.65 Hz, 2H), 2.90 – 2.86 (m, 2H), 2.61 – 2.57 (m, 2H), 2.09 (t, *J* = 11.7 Hz, 2H), 1.75 (d, *J* = 12.6 Hz, 2H), 1.56 – 1.42 (overlapping Boc s + H$^1$ m, 10H), 1.22 (m, *J* = 12.3, 3.89 Hz, 2H); $^{13}$C NMR (101 MHz, methanol-$d_4$) 161.1 (d, *J* = 243.22), 157.2, 130.7 (d, *J* = 4.79), 127.8 (d, *J* = 8.14), 126.5 (d, *J* = 16.06), 123.9 (d, *J* = 3.56), 114.7 (d, *J* = 22.43), 78.4, 58.7, 52.9, 45.3, 36.2, 29.0, 27.3, 25.8; $^{19}$F NMR (376 MHz, methanol-$d_4$) -121.2; UV ($\lambda_{max}$ nm): 264 nm; HRMS (ESI) calc'd for $C_{19}H_{29}FN_2O_2$ [M+H]$^-$: 335.2140, found: 335.2136.

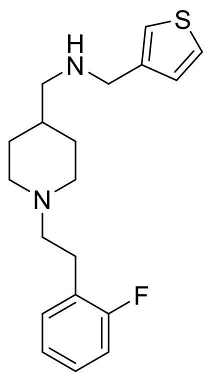

**1-(1-(2-Fluorophenethyl)piperidin-4-yl)-N-(thiophen-3-ylmethyl)methanamine (S9):** *tert*-butyl ((1-(2-fluorophenethyl)piperidin-4-yl)methyl)carbamate (1 equiv., 2.24g) was dissolved in dichloromethane (25 mL). Trifluoroacetic acid (10 mL) was added to the reaction and was stirred for 1 hour at room temperature. Solvent was then removed under reduced pressure, methanol (20 mL) was added and the resulting solution reduced under reduced pressure to facilitate removal of residual trifluoroacetic acid. This process was repeated two additional times. The resulting crude product (0.975 g) was dissolved in a methanol-acetic acid mixture (19:2, 21 mL) with thiophene-3-carbaldehyde (0.95 equiv., 224 mg). pic-$BH_3$ (1 equiv., 225 mg) was then added and the reaction was stirred at room temperature under ambient atmosphere. After two hours, the reaction was judged to completion by TLC analysis (6:2:92 methanol:triethylamine:ethyl acetate). Solvent was then removed under reduced pressure and 10% aqueous hydrochloric acid (21 mL) was added. The aqueous solution was stirred for 0.5 hours at room temperature. The reaction was then cooled to 0 ℃ and aqueous sodium carbonate was added to the reaction until the pH reached approximately 8 (via pH paper). The aqueous solution was then extracted with ethyl acetate (3x, 20 mL). The organic layers were combined and washed with brine (1x, 40 mL), and dried over sodium sulfate. The sodium sulfate was then removed via filtration and the resulting solution concentrated under reduced pressure. The crude product was then purified via flash chromatography with 6:2:92 methanol:triethylamine:ethyl acetate to yield **S9** as a yellow oil (368 mg, 53% yield). $^1$H NMR (400 MHz, methanol-$d_4$) δ 7.39 – 7.35 (m, 1H), 7.29 – 7.26 (m, 2H), 7.26 – 7.20 (m, 1H), 7.13 – 7.09 (m, 2H), 7.09 – 7.01 (m, 1H),3.79 (s, 2H), 3.32 (p, *J* = 6.15, 3.25 Hz, 1H), 3.09 – 3.02 (m, 2H), 2.90 – 2.84 (m, 2H), 2.61 – 2.55 (m, 2H), 2.49 (d, *J* = 6.81 Hz, 2H), 2.10 (t, *J* = 11.80 Hz, 2H), 1.80 (d, *J* = 12.90 Hz, 2H), 1.63 – 1.50 (m, 1H), 1.28 (m, 2H); $^{13}$C NMR (101 MHz, methanol-$d_4$) δ 162.5 (d, *J* = 244 Hz), 141.5, 132.1 (d, *J* = 4.0 Hz), 129.2 (d, *J* = 8.1 Hz), 128.8, 127.9 (d, *J* = 16.2 Hz), 126.7, 125.3 (d, *J* = 4.0 Hz), 123.3, 116.1 (d, *J* = 22.2 Hz), 60.2, 55.6, 54.4, 49.2, 36.7, 31.1, 27.2; $^{19}$F NMR (376 MHz, methanol-$d_4$) δ -121.2; UV ($\lambda_{max}$ nm): 280 nm; HRMS (ESI) calc'd for $C_{19}H_{26}FN_2S$ $[M+H]^+$: 333.1795, found: 333.1791.

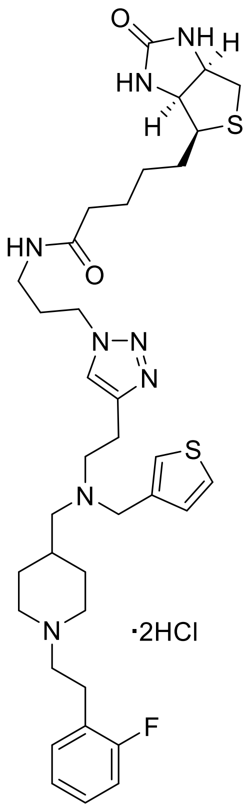

**N-(3-(4-(2-(((1-(2-fluorophenethyl)piperidin-4-yl)methyl)(thiophen-3-ylmethyl)amino)ethyl)-1H-1,2,3-triazol-1-yl)propyl)-5-((3aS,4S,6aR)-2-oxohexahydro-1H-thieno[3,4-d]imidazol-4-yl)pentanamide (S10):** Compound **S9** (1 equiv., 700mg) was dissolved in acetonitrile (50 mL) and dry potassium carbonate (4 equiv., 1.16 g) was added. The reaction vessel was purged with Ar and the solution was heated to reflux under Ar. 4-Bromobut-1-yne (1.2 equiv., 336 mg) dissolved in acetonitrile (5 mL) was then added slowly to the reaction over an hour via syringe pump. The reaction was then cooled to room temperature and concentrated under reduced pressure. The resulting mixture was then dissolved in ethyl acetate (50 mL) and washed with brine (1x, 40 mL). The organic layer was dried over sodium sulfate. Sodium sulfate was then removed via filtration and the resulting filtrate was concentrated solvent under reduced pressure. The resulting crude product (25 mg) was placed in a 3 mL vial and a mixture of 1: 1 mixture of water and *tert*-butanol (0.5 mL : 0.5 mL), was added. After the crude product dissolved under stirring, 5-[(3as,4s,6ar)-2-oxo-1,3,3a,4,6,6a-hexahydrothieno[3,4-d]imidazol-4-yl]-n-(3-azidopropyl)pentanamide (biotin azide, , 25 mg) was added. A freshly-prepared 1 M solution of sodium ascorbate (3.3 µmol, 30 µL) was then added followed by the addition of a catalytic amount of copper (II) sulfate pentahydrate (0.65 µmol, in 10 mL of water). The solution was then stirred at room temperature overnight. The reaction was then diluted with water, cooled with ice, and filtered. The solid was washed with cold water and dried under vacuum. The product was then dissolved in methanol (5 mL) and concentrated hydrochloric acid was added to the solution (1 mL). The solution was then concentrated under reduced pressure. Ethanol (3 mL) was then added and the resulting mixture concentrated under reduced pressure to

azeotropically remove water. This was repeated two more times. The resulting light yellow solid was then dried under vacuum overnight to yield **S10** (4 mg, 15% yield) and was dissolved in ethanol. The product was washed with ethanol until all extra acid was removed. $^{1}$H NMR (400 MHz, methanol-$d_4$) δ 7.84 (s, 1H), 7.70 (dd, *J* = 3.0, 1.3 Hz, 1H), 7.52 (dd, *J* = 5.0, 2.9 Hz, 1H), 7.29 – 7.19 (m, 3H), 7.13 – 6.99 (m, 2H), 4.47 (s, 1H), 4.41 – 4.33 (m, 3H), 4.20 (dd, *J* = 7.9, 4.5 Hz, 2H), 3.67 – 3.59 (m, 2H), 3.44 (t, *J* = 7.4 Hz, 2H), 3.29 – 3.23 (m, 2H), 3.18 – 3.13 (m, 4H), 3.13 – 3.09 (m, 2H), 3.05 – 2.98 (m, 2H), 2.84 (dd, *J* = 5.0, 2.9 Hz, 1H), 2.81 (dd, *J* = 4.9, 3.0 Hz, 1H), 2.61 (d, *J* = 5.5 Hz, 1H), 2.58 (d, *J* = 5.6 Hz, 1H), 2.15 – 2.09 (m, 4H), 2.00 (t, *J* = 6.7 Hz, 2H), 1.70 – 1.63 (m, 3H), 1.59 – 1.52 (m, 4H), 1.37 – 1.32 (m, 3H); $^{13}$C NMR (101 MHz, methanol-$d_4$) δ 174.8, 174.8, 164.7, 161.1 (d, *J* = 244.4 Hz), 142.7, 130.8 (d, *J* = 4.1 Hz) 129.5, 129.3 (d, *J* = 8.5 Hz), 128.7, 127.8, 124.5 (d, *J* = 3.4 Hz), 123.2, 122.8 (d, *J* = 5.1 Hz), 115.2 (d, *J* = 21.8 Hz), 62.0, 61.9, 60.2, 57.4, 56.3, 55.6, 52.7, 51.6, 48.7, 39.6, 36.3, 35.8, 35.4, 35.3, 29.6, 29.5, 28.4, 28.3, 28.1, 27.1, 25.5, 25.4, 23.6, 19.5; UV ($\lambda_{max}$ nm) 248; IR $v_{max}$ ($cm^{-1}$) 3233, 2926, 1640; HRMS (ESI) *m/z* calculated for $C_{36}H_{52}FN_8O_2S_2$ $[M+H]^+$: 711.3633, found 711.3631.

**NMR Spectra**

***N*-(2-amino-1-(3-phenylpropyl)-1H-benzo[d]imidazol-5-yl)-3,5-dichloro-*N*-(prop-2-yn-1-yl)benzamide (S5):**

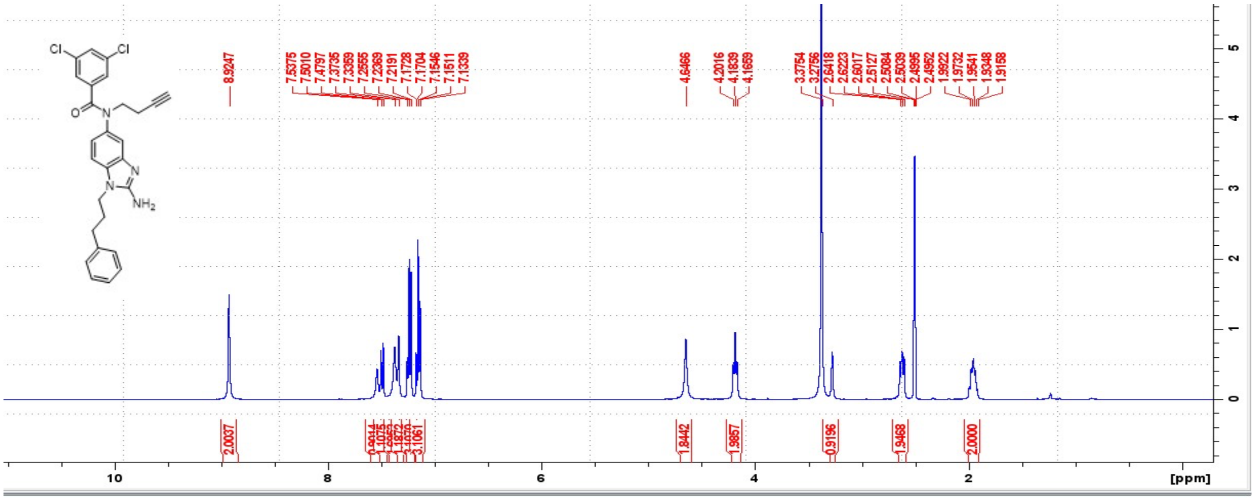


***N*-(2-amino-1-(3-phenylpropyl)-1H-benzo[d]imidazol-5-yl)-3,5-dichloro-*N*-((1-(3-(5-((3aS,4S,6aR)-2-oxohexahydro-1H-thieno[3,4-d]imidazol-4-yl)pentanamido)propyl)-1H-1,2,3-triazol-4-yl)methyl)benzamide (S6):**

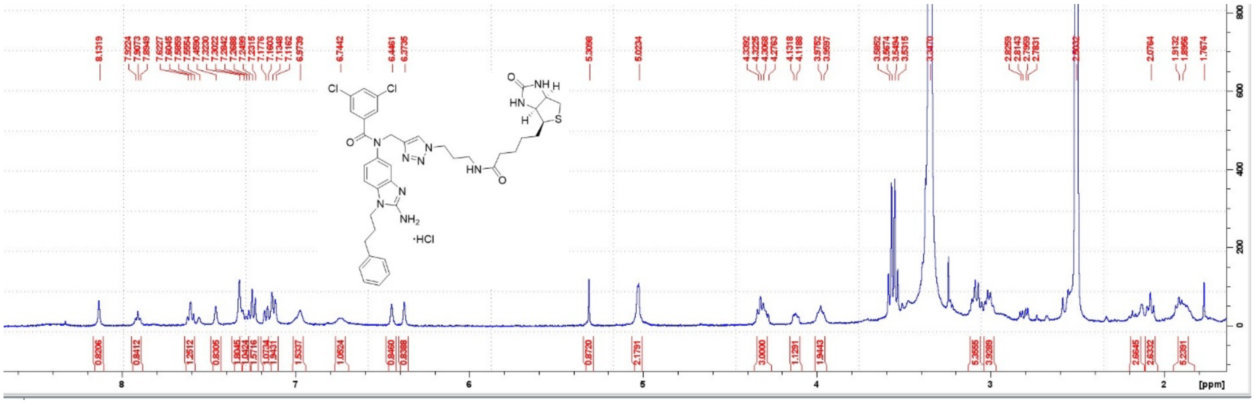

**tert-butyl ((1-(2-fluorophenethyl)piperidin-4-yl)methyl)carbamate (S8):**

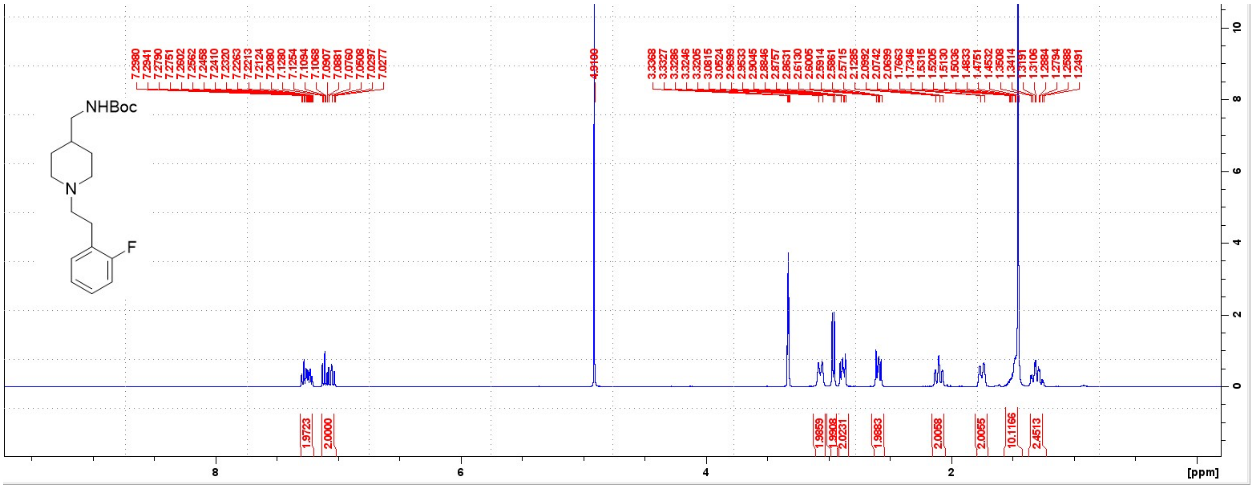

**1-(1-(2-fluorophenethyl)piperidin-4-yl)-N-(thiophen-3-ylmethyl)methanamine (S9):**

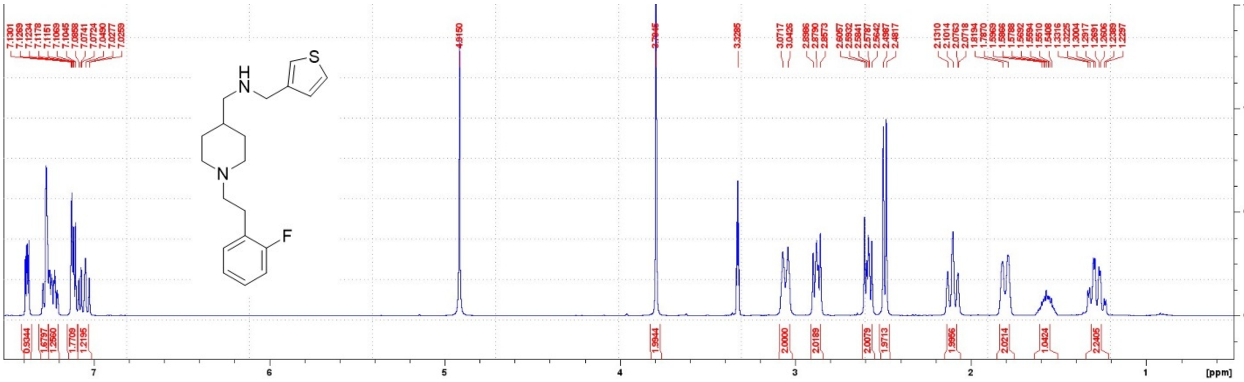

***N*-(3-(4-(2-(((1-(2-fluorophenethyl)piperidin-4-yl)methyl)(thiophen-3-ylmethyl)amino)ethyl)-1H-1,2,3-triazol-1-yl)propyl)-5-((3aS,4S,6aR)-2-oxohexahydro-1H-thieno[3,4-d]imidazol-4-yl)pentanamide (S10):**

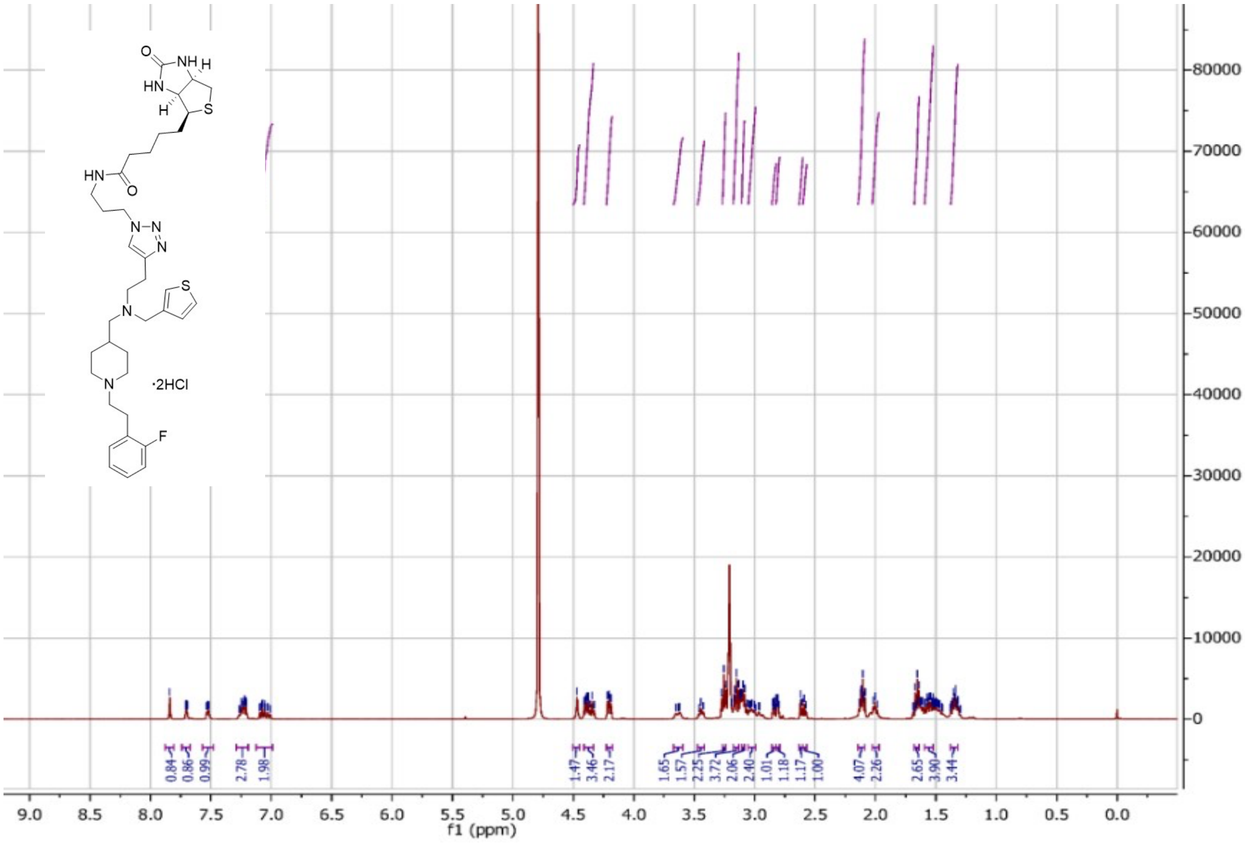



References:

1. T. V. Nguyen, M. S. Blackledge, E. A. Lindsey, D. F. Ackart, A. B. Jeon, A. ObregonHenao, R. J. Melander, R. J. Basaraba and C. Melander, Angew. Chem. Int. Ed., 2017, 56, 3940-3944.
2. Wan, X., Zhang, G., Ge, Z., Narain, R. and Liu, S. (2011), Construction of Polymer–Protein Bioconjugates with Varying Chain Topologies: Polymer Molecular Weight and Steric Hindrance Effects. Chem. Asian J., 6: 2835-2845.
