# Supplementary figures and images for: Mechanisms of antibiofilm compounds JG-1 and M4 across multiple species: alterations of protein interactions essential to biofilm formation
Source: Front Cell Infect Microbiol. 2025 Sep 17;15:1631575. doi: 10.3389/fcimb.2025.1631575 (PMC12484186; doi:10.3389/fcimb.2025.1631575)

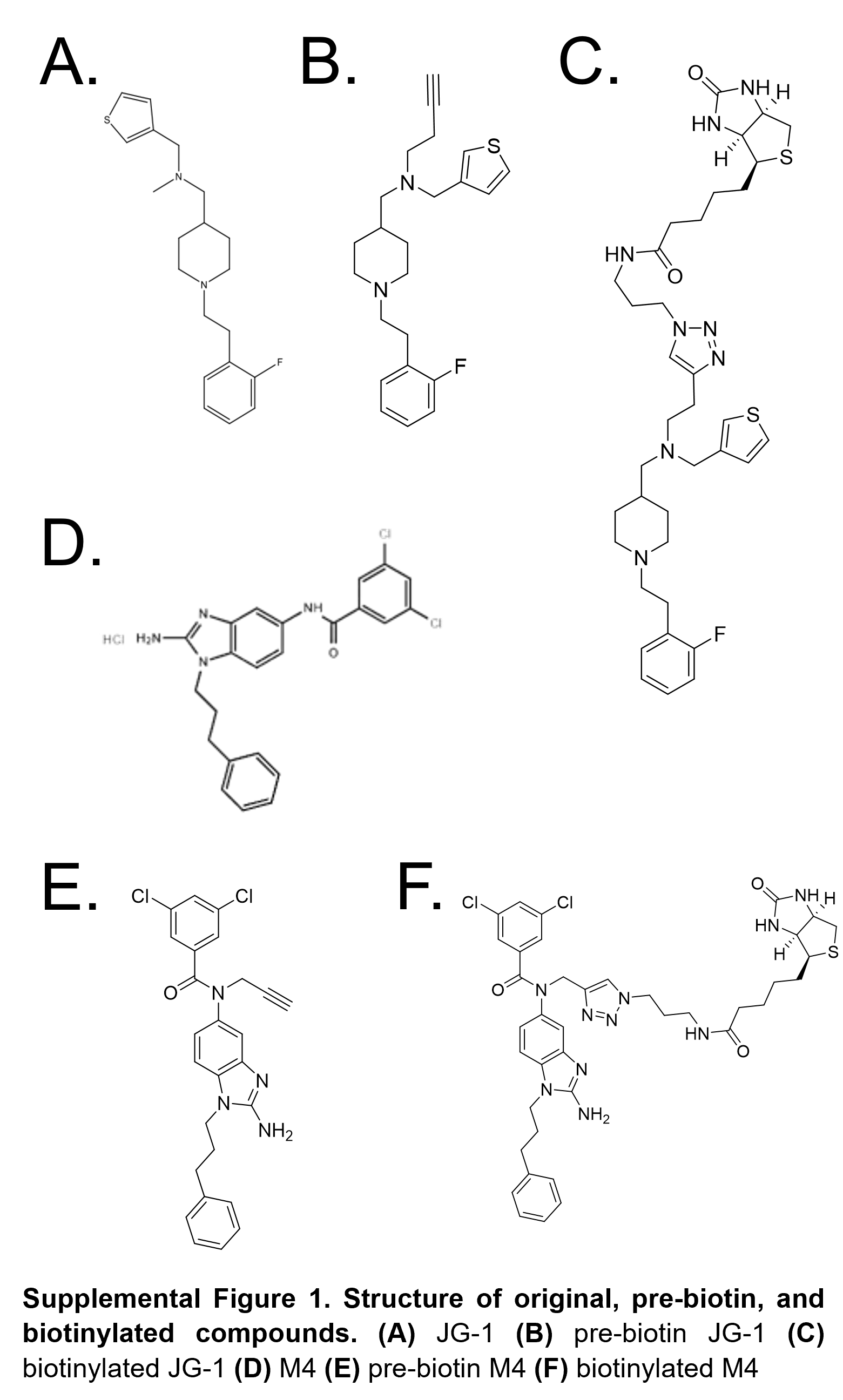

Supplement: Supplementary Figure 1 — Structure of original, pre-biotin, and biotinylated compounds. (A) JG-1 (B) pre-biotin JG-1 (C) biotinylated JG-1 (D) M4 (E) pre-biotin M4 (F) biotinylated M4. [file Image1.png]

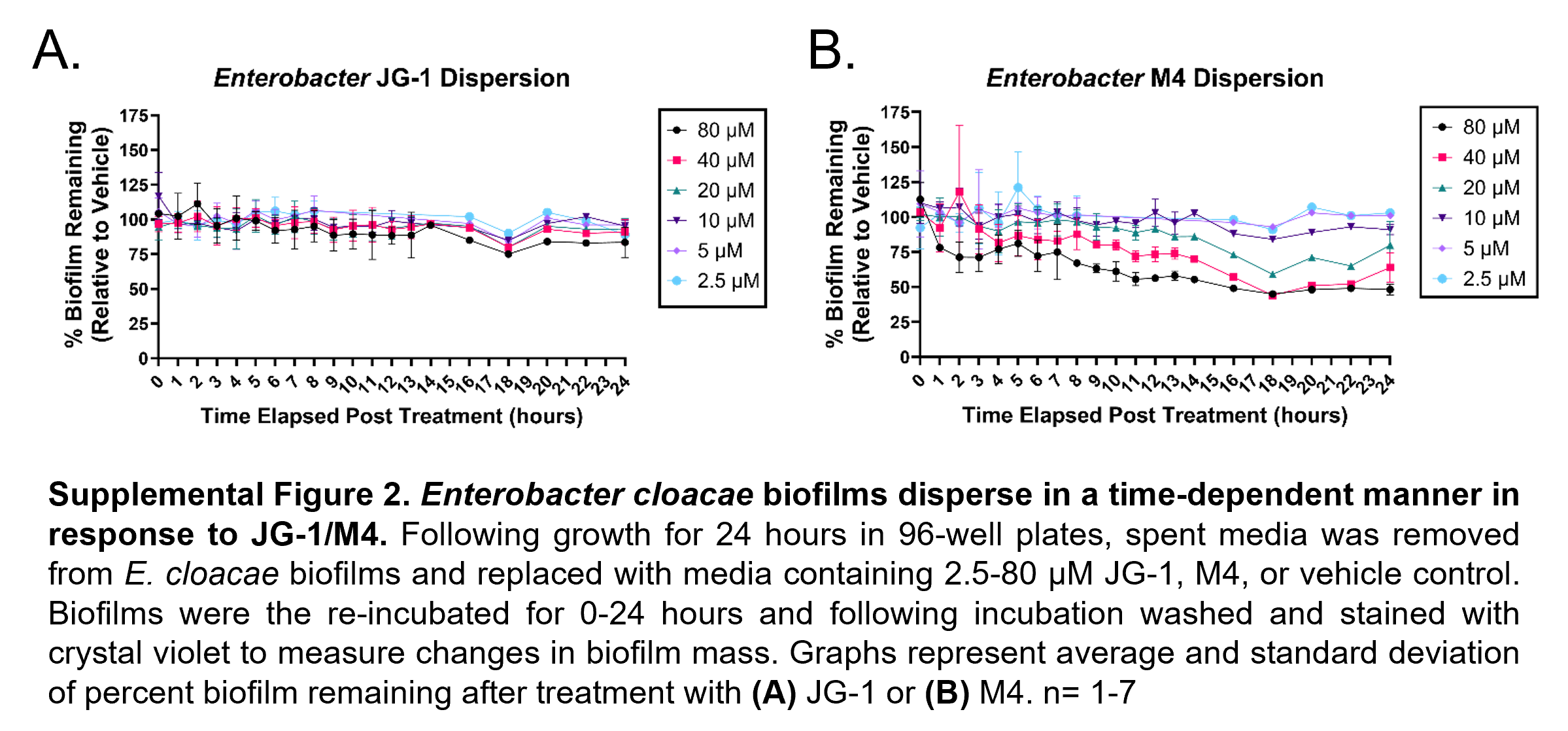

Supplement: Supplementary Figure 2 — Enterobacter cloacae biofilms disperse in a time-dependent manner in response to JG-1/M4. Following growth for 24 hours in 96-well plates, spent media was removed from E. cloacae biofilms and replaced with media containing 2.5-80 µM JG-1, M4, or vehicle control. Biofilms were the re-incubated for 0-24 hours and following incubation washed and stained with crystal violet to measure changes in biofilm mass. Graphs represent average and standard deviation of percent biofilm remaining after treatment with (A) JG-1 or (B) M4. n= 1-7. [file Image2.png]

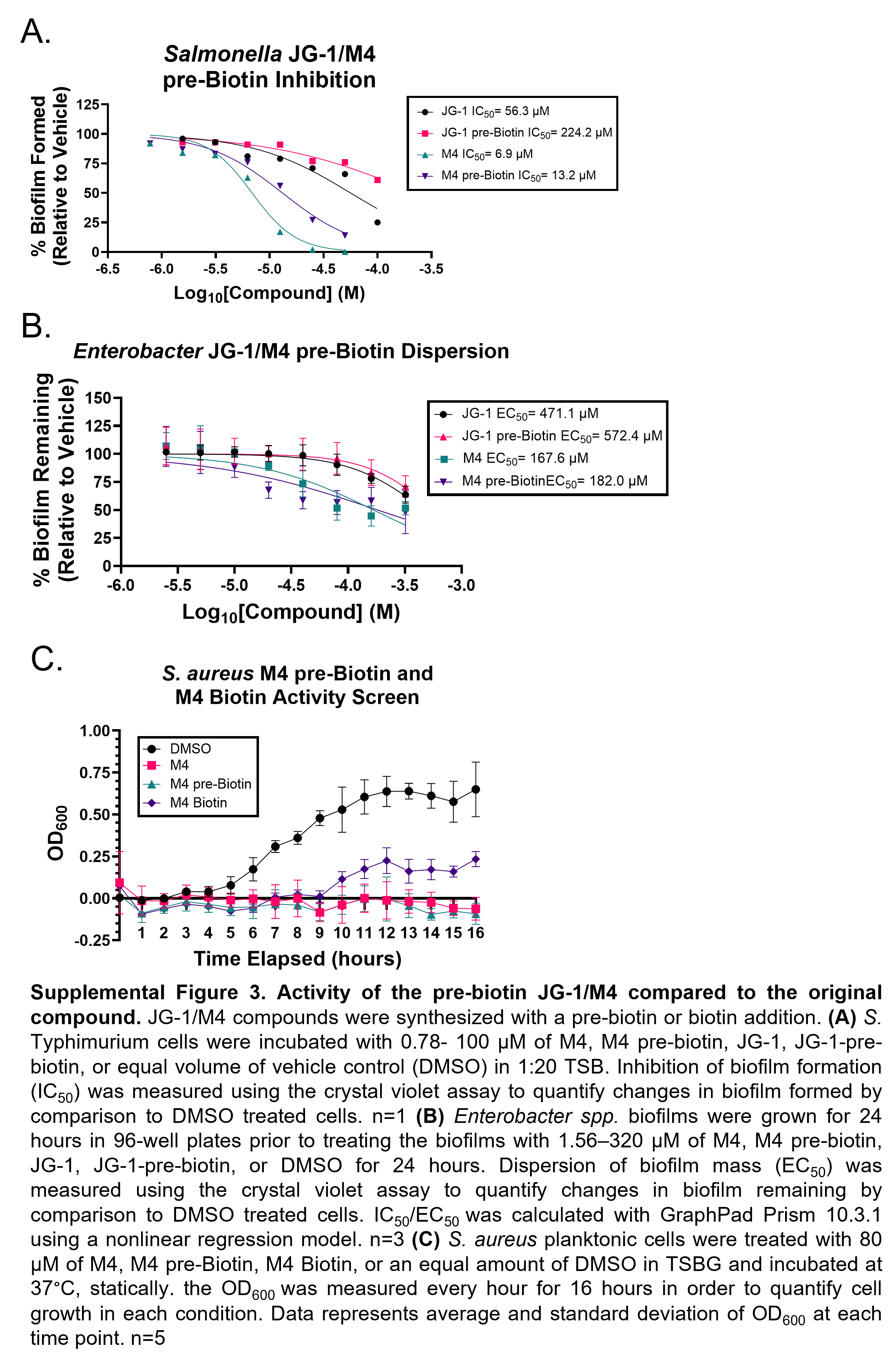

Supplement: Supplementary Figure 3 — Activity of the pre-biotin JG-1/M4 compared to the original compound. JG-1/M4 compounds were synthesized with a pre-biotin or biotin addition. (A) S. Typhimurium cells were incubated with 0.78-100 µM of M4, M4 pre-biotin, JG-1, JG-1-pre-biotin, or equal volume of vehicle control (DMSO) in 1:20 TSB. Inhibition of biofilm formation (IC50) was measured using the crystal violet assay to quantify changes in biofilm formed by comparison to DMSO treated cells. n=1 (B) Enterobacter spp. biofilms were grown for 24 hours in 96-well plates prior to treating the biofilms with 1.56-320 µM of M4, M4 pre-biotin, JG-1, JG-1-pre-biotin, or DMSO for 24 hours. Dispersion of biofilm mass (EC50) was measured using the crystal violet assay to quantify changes in biofilm remaining by comparison to DMSO treated cells. IC50/EC50 was calculated with GraphPad Prism 10.3.1 using a nonlinear regression model. n=3 (C) S. aureus planktonic cells were treated with 80 µM of M4, M4 pre-Biotin, M4 Biotin, or an equal amount of DMSO in TSBG and incubated at 37°C, statically. the OD600 was measured every hour for 16 hours in order to quantify cell growth in each condition. Data represents average and standard deviation of OD600 at each time point. n=5. [file Image3.png]

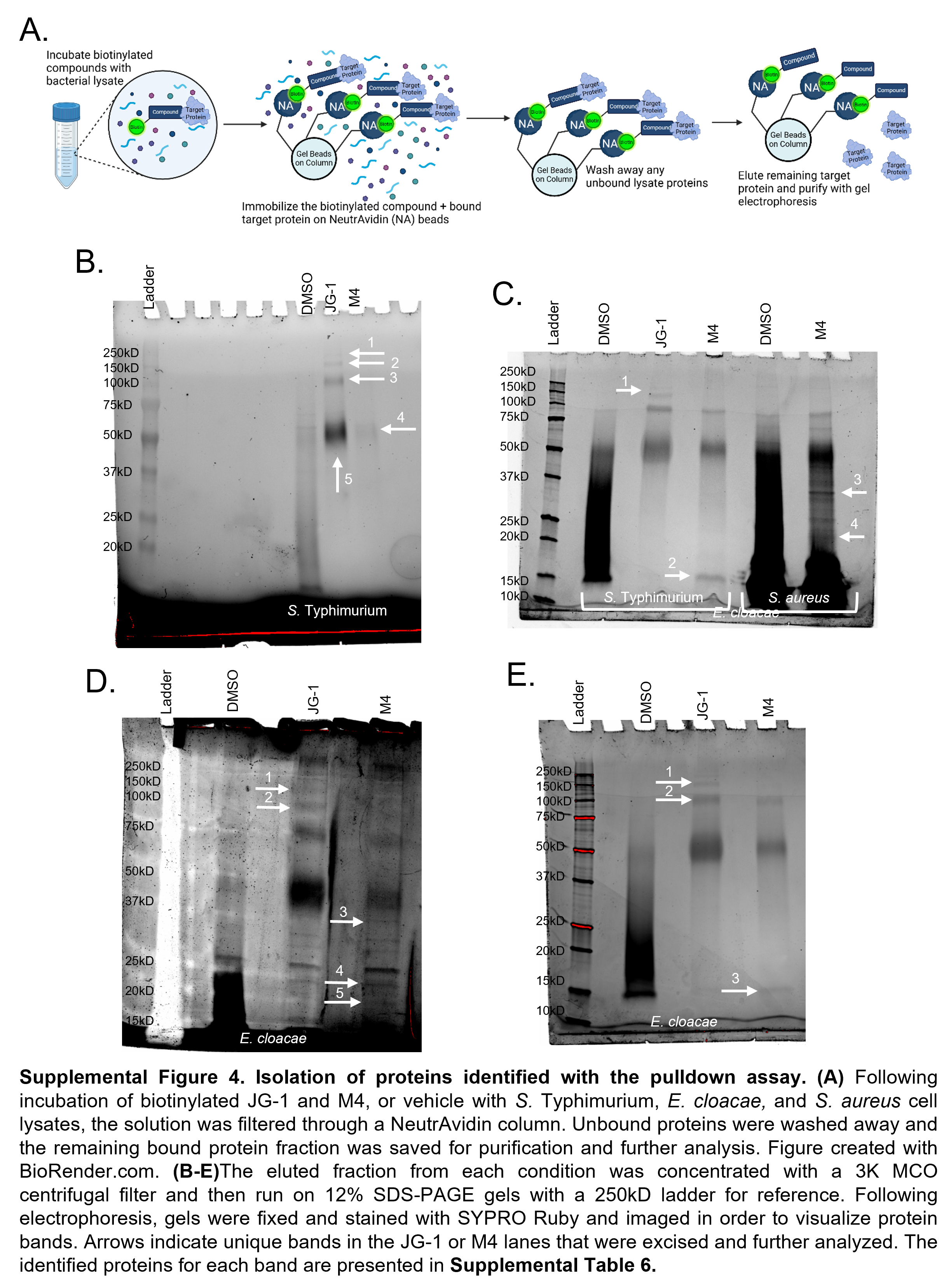

Supplement: Supplementary Figure 4 — Isolation of proteins identified with the pulldown assay. (A) Following incubation of biotinylated JG-1 and M4, or vehicle with S. Typhimurium, E. cloacae, and S. aureus cell lysates, the solution was filtered through a NeutrAvidin column. Unbound proteins were washed away and the remaining bound protein fraction was saved for purification and further analysis. Figure created with BioRender.com. (B-E) The eluted fraction from each condition was concentrated with a 3K MCO centrifugal filter and then run on 12% SDS-PAGE gels with a 250kD ladder for reference. Following electrophoresis, gels were fixed and stained with SYPRO Ruby and imaged in order to visualize protein bands. Arrows indicate unique bands in the JG-1 or M4 lanes that were excised and further analyzed. The identified proteins for each band are presented in Supplementary Table 6 . [file Image4.png]

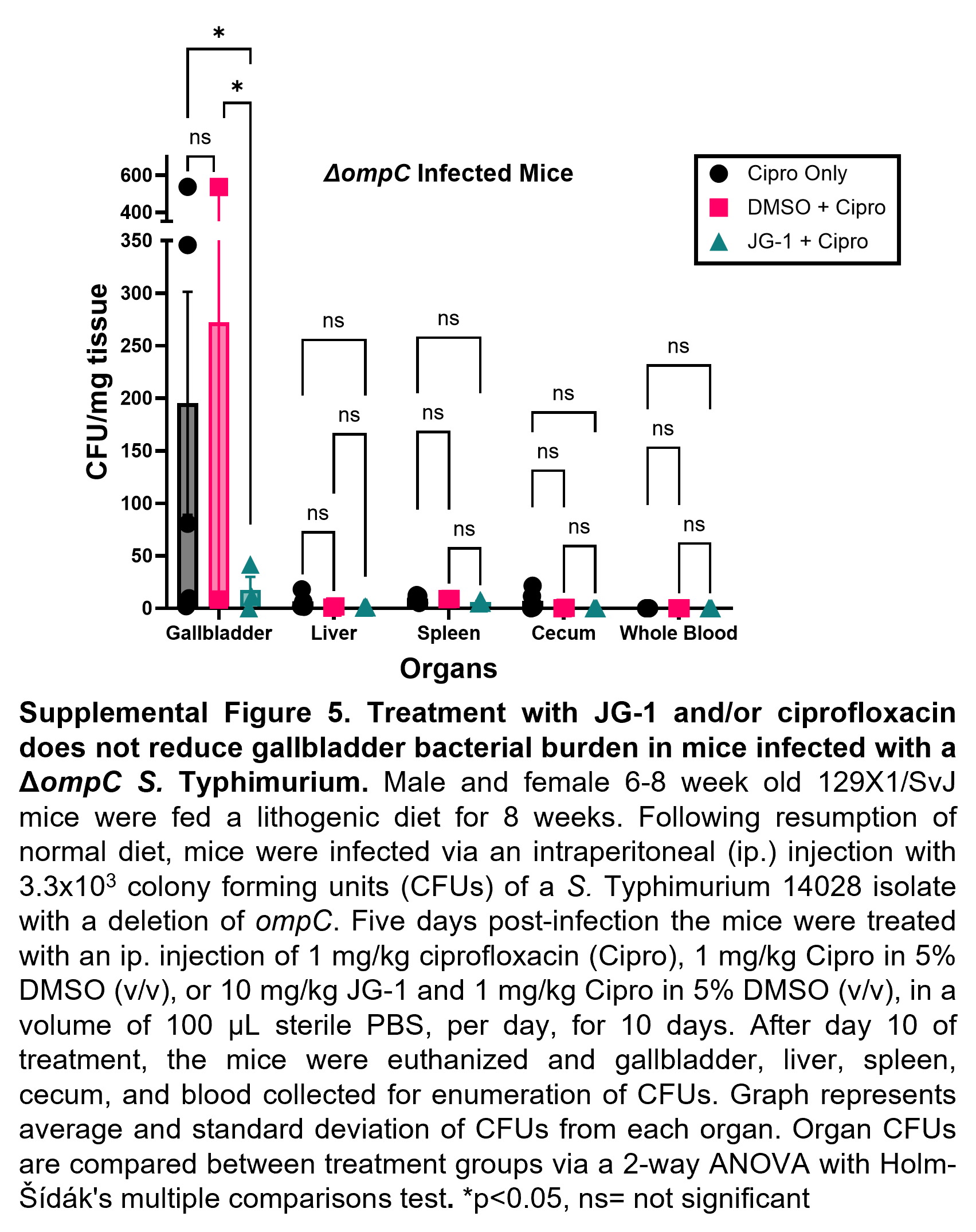

Supplement: Supplementary Figure 5 — Treatment with JG-1 and/or ciprofloxacin does not reduce gallbladder bacterial burden in mice infected with a ΔompC S. Typhimurium. Male and female 6-8 week old 129X1/SvJ mice were fed a lithogenic diet for 8 weeks. Following resumption of normal diet, mice were infected via an intraperitoneal (ip.) injection with 3.3x10^3^ colony forming units (CFUs) of a S. Typhimurium 14028 isolate with a deletion of ompC. Five days post-infection the mice were treated with an ip. injection of 1 mg/kg ciprofloxacin (Cipro), 1 mg/kg Cipro in 5% DMSO (v/v), or 10 mg/kg JG-1 and 1 mg/kg Cipro in 5% DMSO (v/v), in a volume of 100 µL sterile PBS, per day, for 10 days. After day 10 of treatment, the mice were euthanized and gallbladder, liver, spleen, cecum, and blood collected for enumeration of CFUs. Graph represents average and standard deviation of CFUs from each organ. Organ CFUS are compared between treatment groups via a two-way ANOVA with Holm-Šídák’s multiple comparisons test. *p<0.05, ns, not significant. [file Image5.png]
